# Fascicular Topography of the Human Median Nerve for Neuroprosthetic Surgery

**DOI:** 10.3389/fnins.2016.00286

**Published:** 2016-07-01

**Authors:** Ignacio Delgado-Martínez, Jordi Badia, Arán Pascual-Font, Alfonso Rodríguez-Baeza, Xavier Navarro

**Affiliations:** ^1^Department of Cell Biology, Physiology and Immunology, Institute of Neurosciences, Universitat Autònoma de BarcelonaBarcelona, Spain; ^2^Centro de Investigación Biomédica en Red sobre Enfermedades NeurodegenerativasMadrid, Spain; ^3^Department of Human Anatomy and Embryology, School of Medicine, Universidad Complutense de MadridMadrid, Spain; ^4^Department of Morphological Sciences, School of Medicine, Universitat Autònoma de BarcelonaBarcelona, Spain

**Keywords:** neuroprosthesis, neural interface, median nerve, human neuroscience, neuroanatomy, nerve surgery, 3D reconstruction, forearm

## Abstract

One of the most sought-after applications of neuroengineering is the communication between the arm and an artificial prosthetic device for the replacement of an amputated hand or the treatment of peripheral nerve injuries. For that, an electrode is placed around or inside the median nerve to serve as interface for recording and stimulation of nerve signals coming from the fascicles that innervate the muscles responsible for hand movements. Due to the lack of a standard procedure, the electrode implantation by the surgeon is strongly based on intuition, which may result in poor performance of the neuroprosthesis because of the suboptimal location of the neural interface. To provide morphological data that can aid the neuroprosthetic surgeon with this procedure, we investigated the fascicular topography of the human median nerve along the forearm and upper arm. We first performed a description of the fascicular content and branching patterns along the length of the arm. Next we built a 3D reconstruction of the median nerve so we could analyze the fascicle morphological features in relation to the arm level. Finally, we characterized the motor content of the median nerve fascicles in the upper arm. Collectively, these results indicate that fascicular organization occurs in a short segment distal to the epicondyles and remains unaltered until the muscular branches leave the main trunk. Based on our results, overall recommendations based on electrode type and implant location can be drawn to help and aid the neuroprosthetic procedure. Invasive interfaces would be more convenient for the upper arm and the most proximal third of the forearm. Epineural electrodes seem to be most suitable for the forearm segment after fascicles have been divided from the main trunk.

## 1. Introduction

A neuroprosthesis is an artificial system that provides direct communication with the nervous system. Many of such devices have been successfully used for decades in the treatment of hearing impairment or Parkinson's disease, for example. Every year new applications are being validated in clinical settings (Collinger et al., 2013; Strollo et al., 2014), but the use of neuroprostheses to fully replace an amputated hand or recover its function after injury is still not optimal (Borton et al., 2013).

One of most important factors that limit currently the use of hand neuroprostheses is the poor performance. Current hand neuroprostheses allow control for up to 6 degrees of freedom (DoF) (Belter et al., 2013); however, a healthy hand has 23 or more DoF (Santello et al., 2013). The hand is controlled by 27 muscles (Santello et al., 2013) which receive innervation from three nerves: median, ulnar, and radial; each of them containing several fascicles with hundreds of motor axons. To achieve a natural control of the hand movement, the selective function of these three nerves and their fascicles should be properly replaced by the neuroprosthesis. Such selectivity is an unsolved challenge. Communication selectivity can be improved by refining the neural interfacing element (Navarro et al., 2005; del Valle and Navarro, 2013) and the signal processing algorithms (Micera et al., 2011); generally speaking, the more invasive an electrode is, the higher selectivity can be achieved. But undoubtedly, the location of the interface at the end of the implantation procedure is the factor that influences most selectivity.

Currently, the surgical technique for the implantation of a neural interface is not standardized. Implantation of the Utah slant electrode array (USEA) is done by means of a high-speed insertion system that inserts the electrode shafts through the epineurium so they reach the intraneural structures (Ledbetter et al., 2013). Intrafascicular thin film electrodes, such as LIFE or TIME, are manually inserted in human nerves: the electrode shaft is placed longitudinally or transversally through the nerve aided by a suture thread attached to a needle (Yoshida and Stein, 1999; Lawrence et al., 2004; Boretius et al., 2010; Rossini et al., 2010). To increase the chances to find a useful signal, several electrodes are randomly inserted in the nerve (Raspopovic et al., 2010). Only a recent case study describing the insertion of four tf-LIFE in the humeral segment of the median and ulnar nerve has attempted to improve the implantation selectivity by identifying the main nerve trunk after a partial epineural microdissection (Di Pino et al., 2014). Nerve mapping in amputees or in peripheral nerve injuries is not possible using intraoperative neuromonitoring techniques. Thus, the surgeon must exclusively rely on the nerve topography for the implantation. After Sunderland (1945), several studies revised his description of the intraneural fascicle topography of the median, ulnar, and radial nerves, mainly in the context of microsurgical nerve repair (Sun et al., 2009; Barcelo et al., 2013). Only recently the problem has been approached from the neuroprosthetic perspective, trying to describe the fascicles innervating the thenar and lumbrical muscles in the distal median nerve (Planitzer et al., 2014).

This study attempts to provide a topographical description of the human median nerve from the proximal segment in the upper arm to the distal part of the nerve. For this purpose, we first performed a statistical description of the motor branches of the median nerve. Then, we characterized the intraneural fascicular morphology through a 3D reconstruction of different representative histological sections of the median nerve and mapped its correspondence with the target muscles. Finally, we quantified the content of motor fibers in the humeral portion of the nerve, which is a frequent location for neural interfaces for transradial amputees (e.g., Dhillon et al., 2004; Rossini et al., 2010; Raspopovic et al., 2014). The results will serve as guidance for future electrode implantation in clinical settings, as well as for neurosurgical repair of proximal injuries.

## 2. Materials and methods

Eight median nerves from 5 body donors (3 left arms and 5 right arms) were investigated at the anatomy laboratories of Universitat Autònoma de Barcelona, and Universidad Complutense de Madrid. While alive, all donors had given their informed consent to the donation of their bodies for teaching and research purposes. In all, the cause of death did not affect the muscles or nerves in the forearm. The protocol was approved by the Ethical Committee of our institutions.

### 2.1. Anatomical dissection and morphological inspection

**Figure 1:**
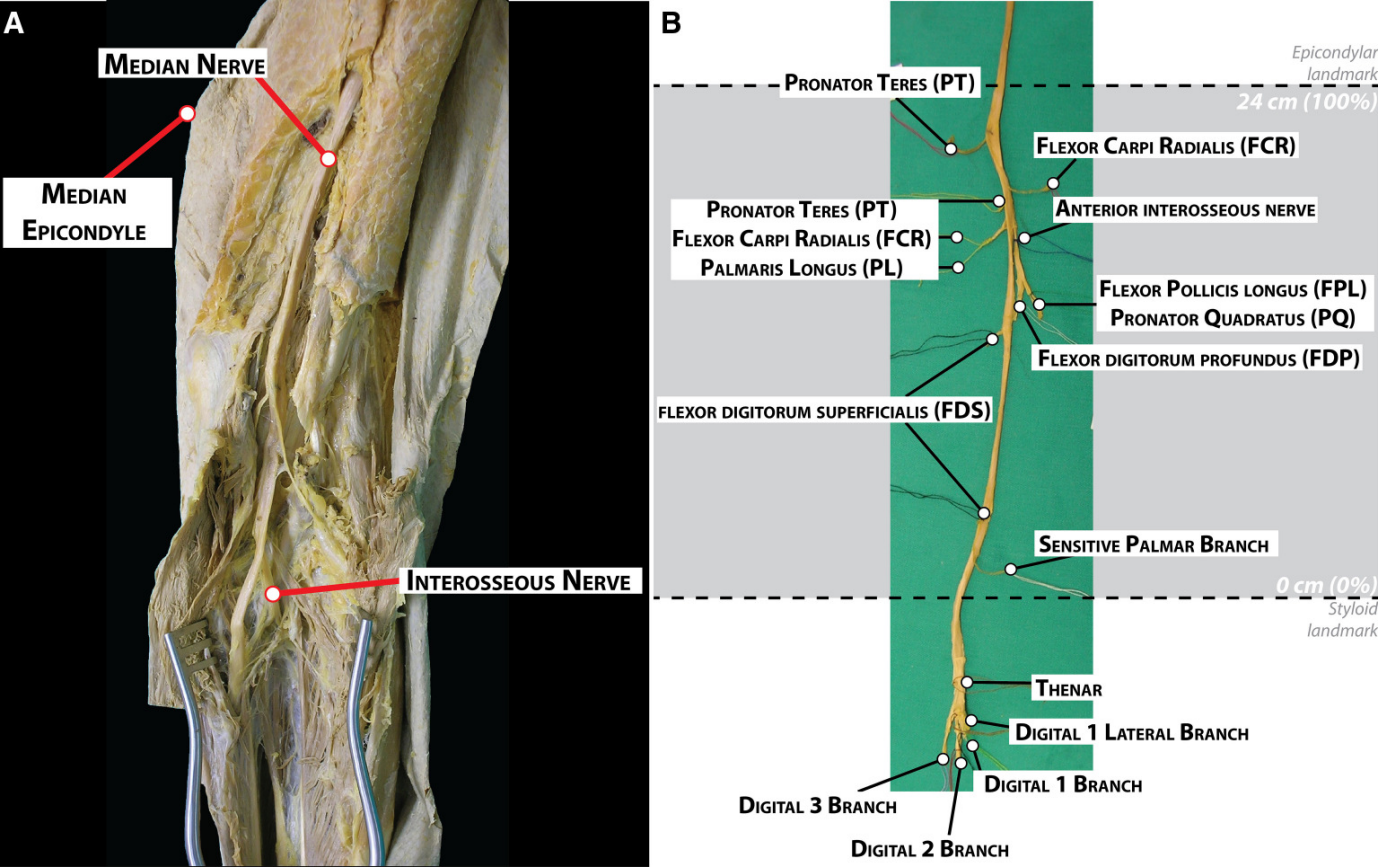
**Dissection and morphological branching description of a human median nerve**. **(A)** Dissection of the medial facet of the arm at elbow level showing the median nerve and some of its branches. **(B)** Harvested median nerve from **(A)** showing the exiting branches to the corresponding muscles. The location of the epicondylar and the styloid landmarks in this nerve is indicated with dashed lines.

Dissection was performed layer by layer through midline ventral incisions in the skin from the axilla to the hand to expose the muscles of the arm, forearm, and hand, as well as the median nerve with its branches (Figure 1A). Branches emerging out the main median trunk were labeled with color threads and tracked to the destination muscle or cutaneous territories for later identification. Nerves to the deep muscles of the forearm were dissected after longitudinal splitting of the flexor digitalis profundus from the fibrous arch. The dissection ended after proper isolation of the digital cutaneous and recurrent branches at the palmar side of the arm. The complete median nerve with its branches was finally harvested (Figure 1B).

During the dissection, the number of primary nerve branches or collaterals of the median nerve, the muscles innervated by these branches and their location were noted. Two locations were labeled on the main trunk: the crossing to a line between the medial and lateral epicondyles at the cubital fossa (epicondylar landmark) and crossing to a line between the radial and ulnar styloid processes at the carpal tunnel (styloid landmark). The length of the forearm was defined as the distance between the epicondylar and the styloid landmarks. This distance was used for size normalization between the subjects (normalized Forearm Distance, nFD, values in percentage,%). The origin of each nerve branch was measured taking the styloid landmark as origin.

To compare the branching pattern between subjects, we calculated the branching probability *P*_*M*_(*x*), of a nerve branch to a given muscle, *M*, at a distance *x* from the styloid landmark (see Supplementary Material). It refers to the proportion of nerve branches that have left the nerve main trunk proximal to a given location with respect to the total number of branches providing innervation for that muscle. Thus, its complement, 1 − *P*_*M*_(*x*), provides an estimate of the probability that a given nerve branch is present in the median nerve trunk at a given location. For neuroprosthetic applications, the complement of the branching probability will indicate the chances for a muscle branch to be in the neighborhood of an electrode implanted in that place.

### 2.2. Choline acetyltransferase (ChAT) immunohistochemistry

Immunohistochemistry labeling of motor fibers by ChAT in nerve cross-sections was done to describe the organization of motor axons in the upper arm segment of the median nerve, where fascicles traveling to the forearm and hand are not segregated yet.

Samples from five nerves were embedded in paraffin and cut on a microtome in 4-μm-thick cross-sections at two levels (nFD = 120% and nFD = 140%). Sections were mounted on silane-coated glass slides, dried overnight, and processed for immunohistochemical detection of ChAT as described before. (Lago and Navarro, 2006; Badia et al., 2010). Briefly, after treatment with sodium citrate buffer (10.2 mM, pH 6.1) for 20 min at 95°C for antigen retrieval, the slides were incubated with 3.3% H_2_O_2_ to inhibit endogenous peroxidase and then the nerve sections were blocked with horse serum (10%) and BSA (3%) in Tris-phosphate buffer (TBS) with 0.3% in Triton X-100 for 1 h at 4°C. They were incubated for 3 days with goat anti-cholineacetyltransferase primary polyclonal antibody (ChAT; 1/75; AB144P; Millipore), to identify motor axons. The primary antibody was diluted in TBS containing 0.3% Triton X-100, 5% serum. After washes with TBS containing 0.3% Triton X-100 (7 × 10 min), sections were incubated for 24 h at 4°C with biotin conjugated horse anti-goat IgG antibody (Vector Laboratories, Peterborough, UK), diluted 1:200 in TBS with 0.3% Triton X-100. To visualize the presence of ChAT the ABC-peroxidase kit with DAB-nickel (Vector) was used. Slides were dehydrated through a series of alcohols and mounted in DPX (Sigma Aldrich, St. Louis, MO, USA). All the samples were subjected to the immunohistochemical procedure in pairs, with one sample of each pair not incubated with the primary antibody to act as a negative control.

Images of the whole median nerve were acquired at either 4 × or 10 × magnification and merged to create the full section and imported into Fiji software (Schindelin et al., 2012) for automatic detection of axons. Structures susceptible to be axons were labeled using the “Analyze Particles” command on the binary mask of the image obtained by Bernsen's local thresholding method (Bernsen, 1986; Sezgin and Sankur, 2004) on a previously corrected one using a contrast limit adaptive histogram equalization (CLAHE) (Pizer et al., 1987). For ChAT^+^ axon identification, the binary mask was created by applying a maximum entropy threshold on the original picture (Sahoo et al., 1988). Structures with a pixel area higher than 5 standard deviations of the mean pixel area of all the structures identified in the section were considered as artifact and discarded from the analysis.

### 2.3. 3D nerve reconstruction

Nerve reconstruction was done to represent in a 3D model the fascicular topography of the median nerve at different regions, and to compare its features between subjects.

One median nerve was split into 11 blocks (detailed in **Figure 5**), subsplit each into segments (block III, 6 segments; block VII, 5 segments; all the others, 3 segments), and sectioned in 950 slices of 25 μm-thickness and 250 μm separation to make a 3D reconstruction. The first block (I) was collected at the styloid landmark. The following 3 blocks (II-IV, distal forearm blocks) were obtained between the styloid landmark and the end of the region where the main muscular branches exit, namely the region described by the 95% confidence interval for the average branching point of the interosseous nerve. The next blocks (V-VIII, proximal forearm blocks) were gathered precisely in that region, right before to the exit of each one of the main branches. The final blocks (IX-XI, upper arm blocks) were obtained from the upper arm trunk. 16-bit digital images of each individual section were acquired using an Olympus C-3050 camera attached to an Olympus microscope (BX51) at 2.0 × magnification and a resolution of 2556 × 1917 pixels. Images were then processed using OpenCV 3.0.0. Otsu's automated thresholding algorithm was used to identify the individual fascicles and generate the initial binary masks. When detection was unsatisfactory, resulting masks were manually verified and corrected. A total of 18,122 fascicles were identified. Masks were then imported in Houdini Apprentice non-commercial edition 13.0.665 (Side Effects Software, Canada) running in a Linux system (kernel 3.18.7) for producing a 3D model. Custom scripts were done in python 2.7.10 using the software toolkit. Firstly, the binary masks were loaded in the compositing editor. Fascicles were labeled using the connected-component method and exported to the “Objects context” converted into polygonal primitives to generate the geometry masks. Next, each slice in a segment was aligned to the previous slice using a iterative closest point algorithm. Then, the centroid of each fascicle in the slice was connected to the nearest centroid of the fascicles of the previous slice and the following one through a straight line. Those lines were connected one after the other to reconstruct the skeleton of the fascicle and create the 3D model. An average Z-projection of the 3D model for each segment was obtained as well.

Descriptive values for each fascicle were obtained from the 3D model. For each single fascicle, the area and perimeter, eccentricity of the shape, position of the centroid, and the distance between the fascicle centroid and the centroid of the geometry mask were measured. Additionally, the area of the ellipse best containing all the fascicles within was calculated to represent the epineural space.

### 2.4. Cluster analysis of fascicle topography

In order to visualize the differences in the pattern of distribution of the fascicles in the different nerve segments, a clustering analysis was done using SPSS 22.0 (IBM Corp., USA). Initially, an agglomerative clustering of the log_2_ values of area and distance of the fascicle to the slice centroid using a nearest neighbor method of Euclidean distances was performed so that the number of clusters, *k*, could be guessed using the Elbow criteria. *k*-means clustering was performed at this particular *k*-value as well as other limiting values. The *k*-value that provided a higher reduction of percentage of variance was chosen. Specifically, *k* = 3 was found satisfactory for both attributes, resulting in 3 categories for fascicle area (A, B, and C) and other 3 for distance between the fascicle centroid and the centroid of the geometry mask (0, 1, 2). Fascicles were accordingly grouped into 9 categories (A0,…,C2).

### 2.5. Statistical analysis

Statistical analysis was done using SPSS 22.0 (IBM Corp., USA). Values are shown as mean ± S.E.M., unless otherwise indicated. The Kolmogorov-Smirnov's test was used to check for normality, prior to perform ANOVA analysis. When a non-normal distribution was found, the Mann-Whitney's test was used instead. *p*-values of 5% or less were considered as statistically significant.

## 3. Results

### 3.1. Origin and exit points of median motor branches

**Figure 2:**
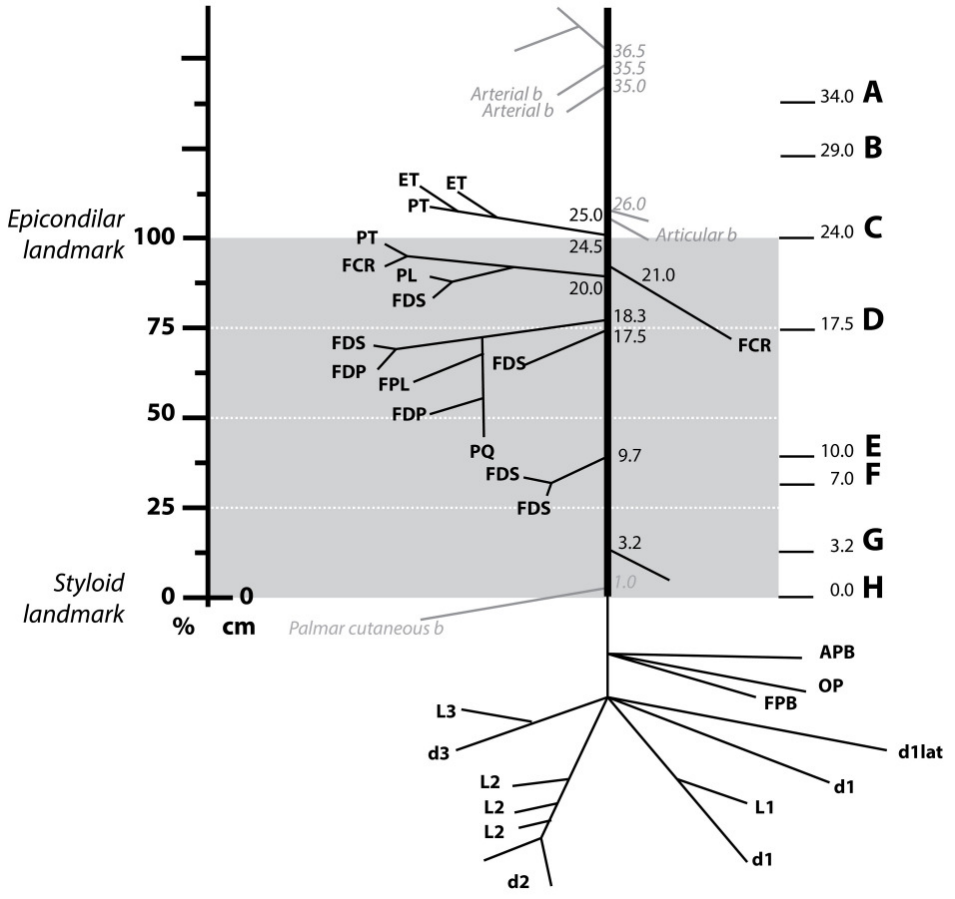
**Distribution of the muscle branches of the median nerve at the forearm**. Schematics showing the origin and destination of a median nerve of the five harvested (Figure S1) with the distances from the styloid landmark to the exit point next to it in cm. The gray area indicates the region between the epicondilar landmark and the styloid one used for normalization of distances (normalized forearm distance, nFD). The vertical axis on the left shows the values of the nFD in percentage. The letter A–H on the right side indicates the level at which the respective slice from **Figure 4** was obtained. Abbreviations in text.

The branching pattern of the median nerve in the forearm was analyzed in five fixed samples (three belonging to the left arm and two to the right arm of four subjects in total, Figure 2 and Figure S1). Despite the high variability, certain similarities were observed (see Table S1). The total length of the samples was 71.3 ± 1.2 cm, divided into the upper arm (29.1 ± 0.5 cm), forearm (25.6 ± 0.5 cm), and hand segments (16.6 ± 0.7 cm). The forearm distance was used for normalizing the size differences between subjects (normalized forearm distance, nFD). An average of 9.2 ± 1.1 branches, sprouting from the main median nerve trunk were identified. From them, 71.0 ± 4.2% (6.4 ± 0.4 branches) terminated in one muscle and were considered muscular branches. They were tracked and found to innervate the following seven muscles: pronator teres (PT), flexor carpi radialis (FCR), palmaris longus (PL), flexor digitorum superficialis (FDS), flexor digitorum profundus (FDP), flexor pollicis longus (FPL), and pronator quadratus (PQ). All but the recurrent branch of median nerve originated in the forearm segment. In one sample, however, one of the muscular branches of the PT exited together with the sensory branches for the proximal radio-ulnar joint at the elbow (articular branches) 5 mm proximally to the epicondylar landmark. Only one sensory branch, the palmar cutaneous branch, was identified in each sample. Additionally, in three samples, several articular branches innervating the proximal radio-ulnar joint (2.0 ± 0.4 branches, *n* = 5) were recognized. In one case the sympathetic branch innervating the brachial artery could be identified.

In all specimens, the first muscular branches were providing the innervation to the PT. The PT receives two branches from the median nerve, commonly named superior and inferior nerves of the PT. In two samples, however, the inferior nerve of the PT was either missing or replaced by fibers coming through the interosseous nerve. The superior nerve of the PT was found to arise more proximal, at the level or below the epicondyle landmark (98.4 ± 2.5% of nFD). In one case, this branch also served fibers to the epitroclear muscles (ET). In other single case, the FCR and PL received innervation from the superior nerve of the muscle PT. The inferior nerve of the PT was clearly identified in two of the three samples as the next distal branch (88.2 ± 3.9% of nFD, *n* = 3). In one of them, however, a branch targeting the FCR originated earlier, between the superior and inferior nerve of the PT (nFD = 87.5%, *n* = 1).

The interosseous nerve was identified in all cases, originating as the next distal branch to the PT nerves (81.0 ± 3.0% of nFD, *n* = 5). This nerve provided branches to the FDP, FDS, PQ, and, in all the cases but one, to the FPL muscles. The interosseous nerve targeted the FCR in three cases. Interestingly, innervation originating from a more proximal branch (i.e., superior nerve of the PT) was observed in the other two samples. Finally, recurrent branches for the ET originating in the interosseous nerve were identified in two cases.

After the interosseous nerve, a variable number of branches (1.8 ± 0.4 branches, *n* = 5) targeting the FDS sprouted out at a nFD of 45.80 ± 4.3%, *n* = 5. Lastly, fibers forming the palmar cutaneous nerve exited at a nFD of 16.5 ± 6.6%, *n* = 5.

**Figure 3:**
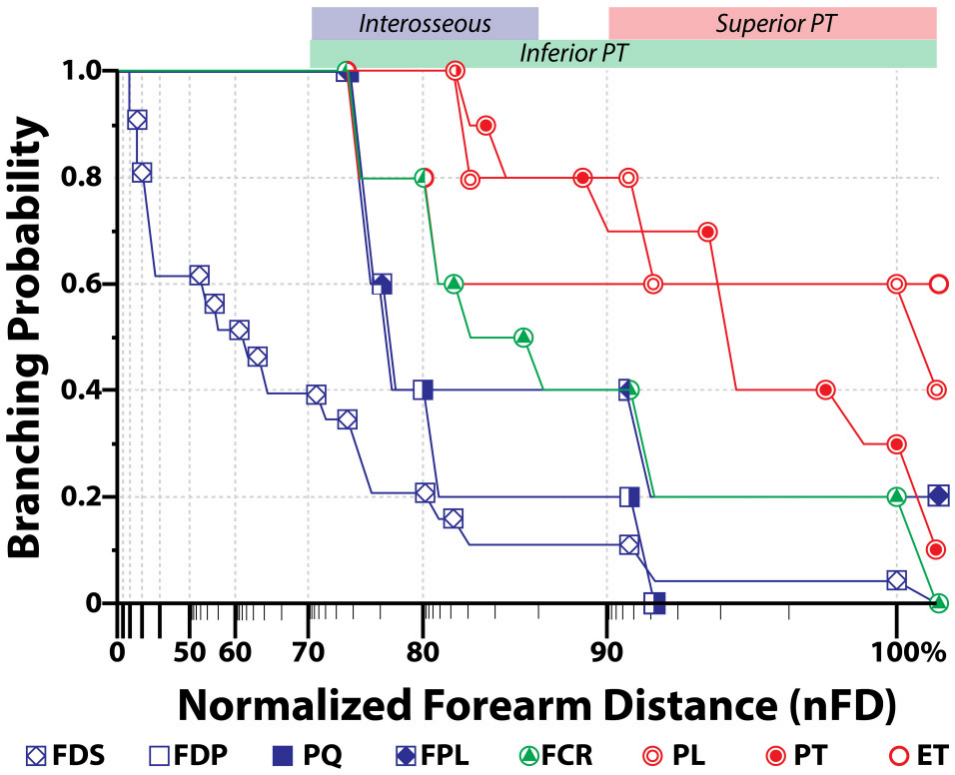
**Branching probability for each muscular branch along the median nerve**. Average probability (*N* = 5) for a muscular branch of the median nerve to exit the main trunk in relationship to the normalized forearm distance, where 0% is the styloid landmark and 100% is the epicondylar one. Distance is in log-scale. Color bands on the top axis represents the 95% confidence interval for the exit position of the indicated nerve branch.

In cases of transradial amputations, the median branches present with marked atrophy, which makes it difficult the electrode implantation procedure (Dhillon et al., 2004). A further complication for the dissection is the high intersubject variability of their location. Here we found that the 95% confidence interval for the average branching point was between 76.67% and 89.32% of nFD (*n* = 5) in the case of the interosseous nerve, 71.42% and 104.98% of nFD (*n* = 3) for the inferior PT, and 91.45% to 105.34% of nFD for the superior PT (*n* = 5, see Figure 3 and Table S1). As consequence, the surgeon has no reliable clues to aid during the procedure and the implantation is done blindly. In order to compare among all the samples the exit points of the nerve branches that innervate a certain muscle, *M*, we calculated the branching probability, *P*_*M*_(*x*). As shown in Figure 3, the branching probability rapidly increased in the most proximal third of the forearm. The *P*_*M*_0.5__-distance, at which the chances that a fascicle has branched out are 0.5, was 110.5 ± 11.1%, 104.5 ± 11.4%, and 95.7 ± 3.4% of the nFD for the PL, ET, and PT, respectively; meaning that these three nerves leave the nerve trunk in most of the cases even before reaching the forearm. But after this point, the branching probability for these fascicles grew only slowly. The fascicles that leave the median trunk within the interosseous nerve presented a *P*_*M*_0.5__-distance inside the confidence interval of this nerve (84.0 ± 4.5% of the nFD for the FCR and 78.5 ± 3.1% of the nFD for the FDP, FPL, and PQ). The branching probability of the PQ and FDP increased abruptly shortly before this point but the probabilities for the FPL and FCR increased only steadily from the segments prior to the epicondyle. Finally, for the FDS the *P*_*M*_0.5__-distance was 63.6 ± 8.3% of nFD. Here the probability increased more uniform from proximal to distal portions of the forearm.

From the defined branching probability, the complement value, i.e., 1 − *P*_*M*_(*x*), can be calculated. This value indicates the probability for a fascicle innervating a certain nerve to be still in the main nerve trunk, which has an important meaning when deciding where in the nerve the electrode will be implanted. On sight of that, we observed that from the segment defined by the 95% confidence interval of the interosseous nerve and more distally, it was unlikely to find fascicles for other forearm muscles apart from the FDS. Within the region of the 95% confidence interval of the superior nerve of the PT, fascicles innervating the PT, PL, FCR, FPL, PQ, FDP, and FDS are likely found in the median nerve, which means that this segment would be the most suitable for electrode implantation.

### 3.2. Intraneural topography of the median nerve

**Figure 4:**
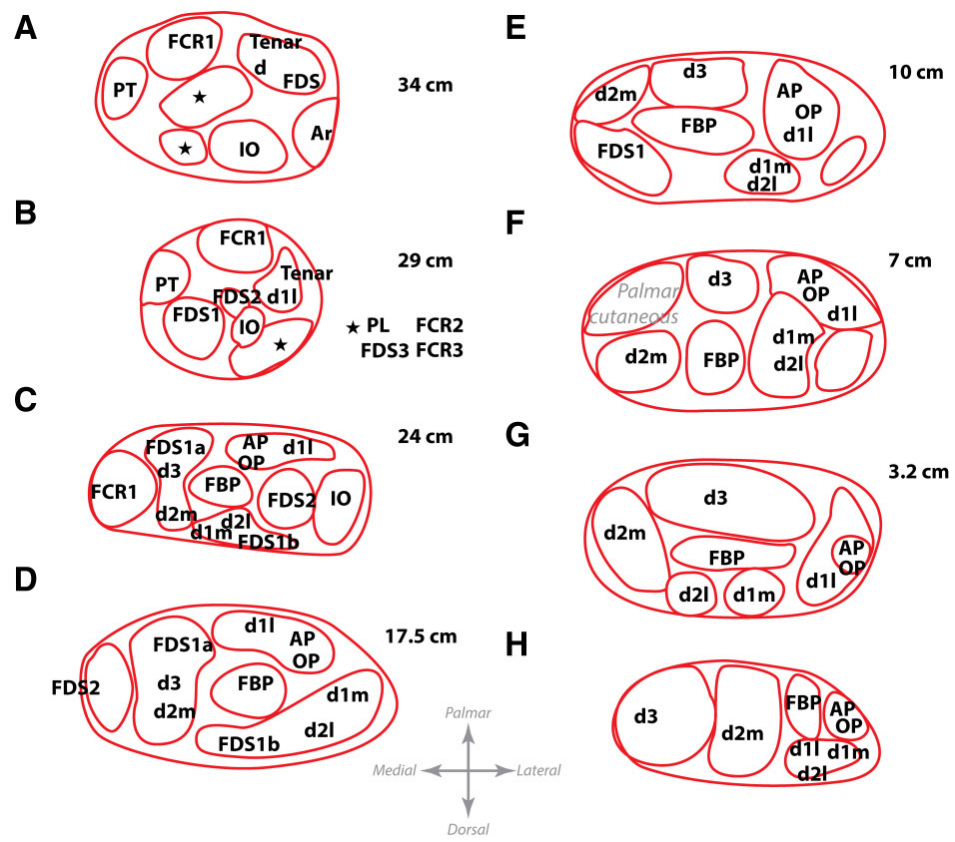
**Location of the fascicles innervating the main muscles of the forearm at different levels**. Letters A–H show from proximal to distal the different levels at which the map of one harvested nerve was obtained. These levels correspond to the same locations as letters A–H in Figure 2. Distances, in cm, from the styloid landmark to the level of each slice are shown besides. Abbreviations in text.

We continued by characterizing the fascicular content at different levels to understand how the muscular branches organize inside the median trunk. For that, we collected histological sections of the segments immediately proximal to a branching point in one of the nerves, noting down the location of the branch. In such way, we mapped the fascicular groups seen in the nerve cross-sections of a segment with the muscular destination of the fascicle (Figure 4). We observed that the fascicles remained in a compact configuration close to the median nerve axis practically until they reached the exit point and branched out toward the corresponding muscle. The division of fascicles into the final branches did not happen until the exit point. Interestingly, the location of the fascicles innervating the distal muscles of the forearm and hand appeared to change during the whole trajectory, presumably because they might accommodate to the earlier exit of the more proximal fascicles, but they did not move far from the central region. After the interosseous nerve and the main core of the FDS bundles departed, the distribution of fascicles for distal muscles remained stable. These observations indicate that fascicles closer to the main axis are those most likely carrying information for the intrinsic muscles of the hand. The fascicles innervating the muscles of the forearm are mostly located at the dorsal aspect of the median nerve surrounding those of the hand. This distribution is important to be considered when choosing a location for implantation of a nerve interface for hand dexterity.

**Figure 5:**
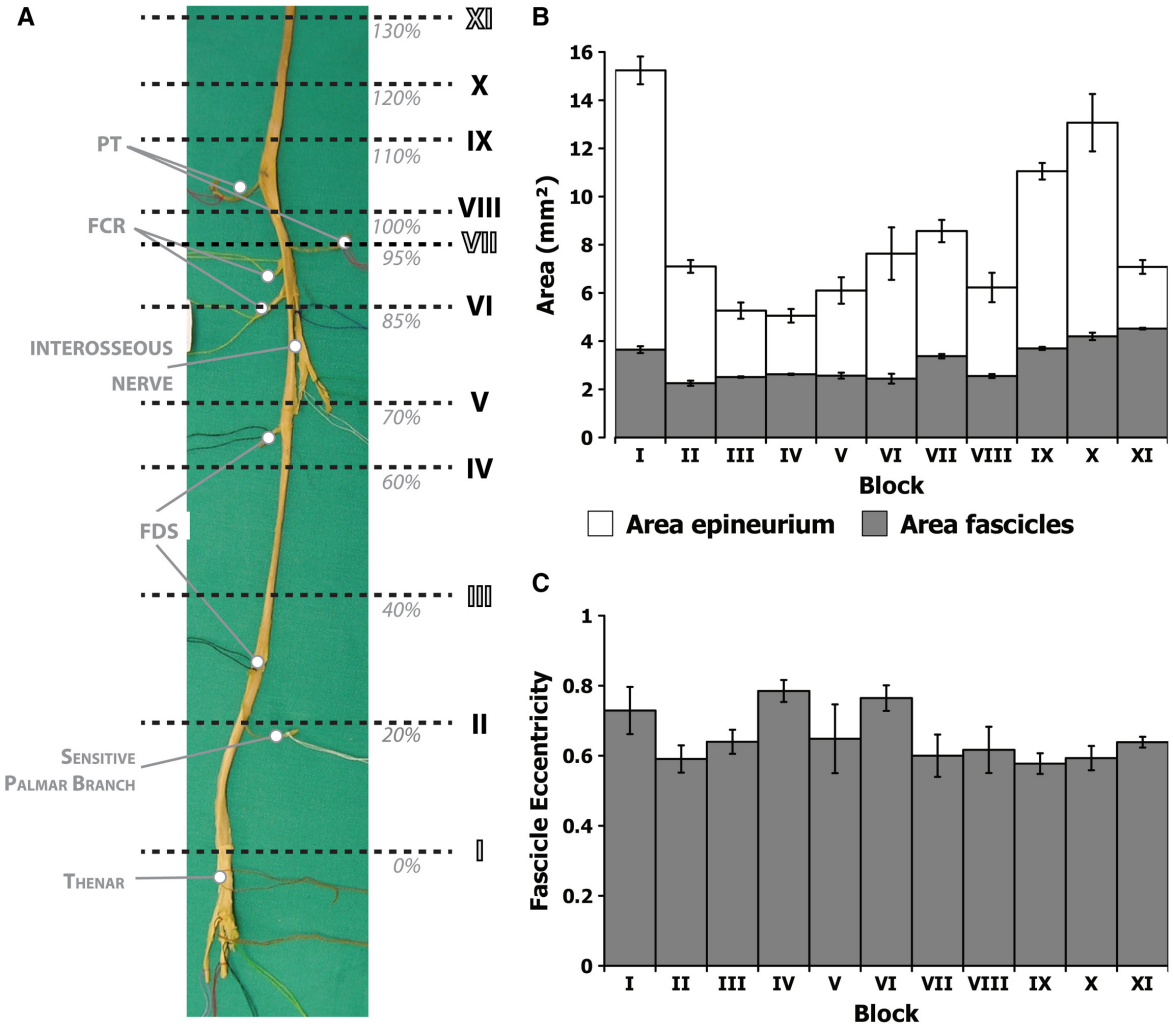
**Morphometric values corresponding to the different block levels of the median nerve**. **(A)** Photograph of the harvested nerve showing the locations of each of the blocks in normalized distances from the styloid landmark (nFD). **(B)** Average area of the epineurium region (white) and the endoneurium (gray) for each of the segments. **(C)** Average eccentricity, i.e., circularity, of the fascicles in each of the segments.

**Figure 6:**
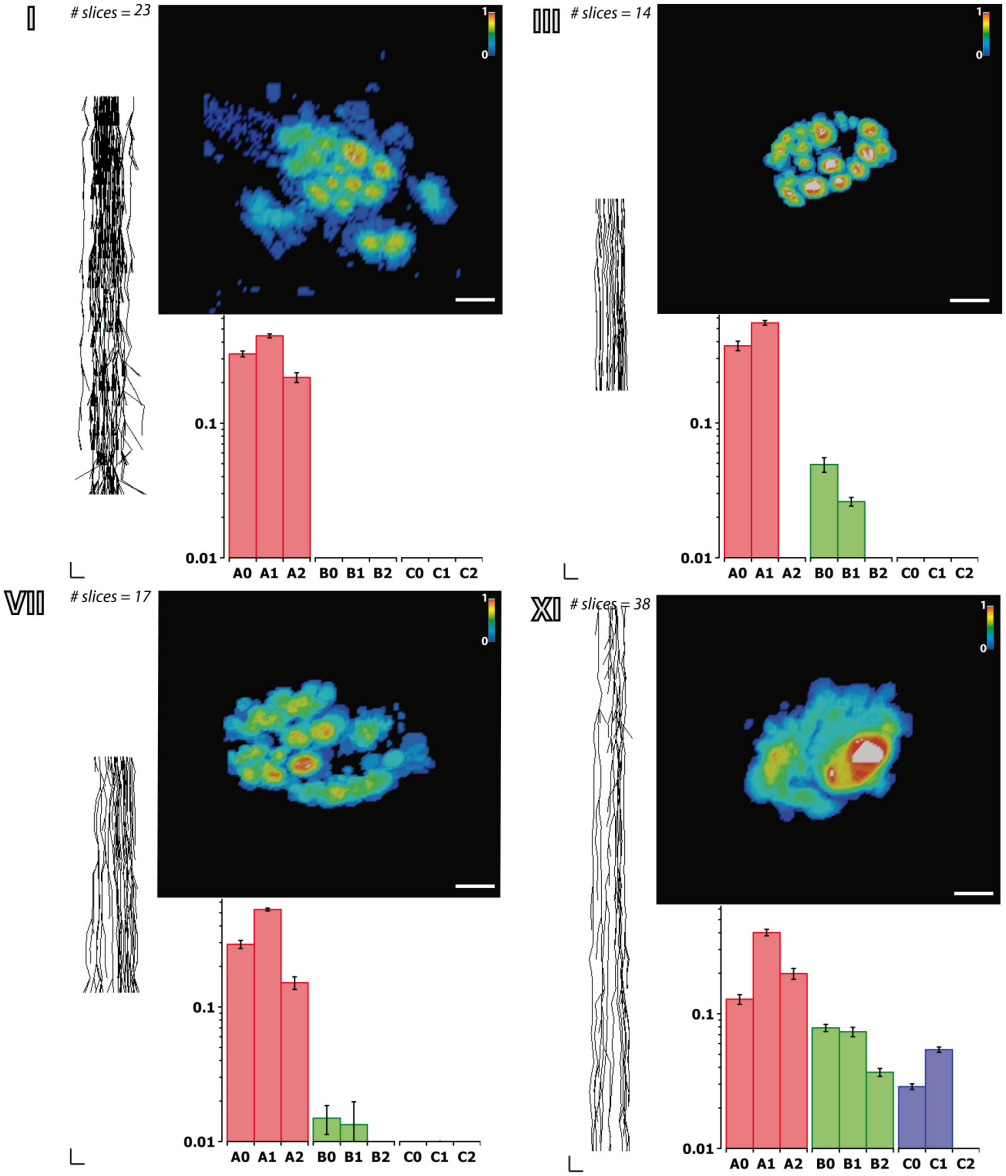
**Fascicular morphology of the median nerve at forearm and upper arm levels**. Analysis of the fascicular morphology at one segment of four different block levels (I, III, VII, and XI) showing each three frames. The element on the left is a planar projection of the 3D reconstruction of the nerve segment with the number of slices used for its calculation (vertical scale = 250 μm, horizontal scale = 1 mm). The top right element represents the probability distribution of the fascicles at each block level along the nerve using a color jet map gradient where warmer hues indicate less variation along the nerve longitudinal axis (vertical color bar, from 0 to 1; horizontal scale bar, 1 mm width). The bottom graph shows the frequency distribution of the fascicles grouped according to the clustering parameters of size (A, B, and C; red, green, and blue, respectively) and the distance to the nerve axis (0, 1, 2) as shown in **Figure 7**. Block levels are depicted in Figure 5.

From the results, it can be hypothesized that bundles carrying information for thenar and intrinsic muscles of the hand may be located in the larger fascicles close to the nerve axis, since they do not leave the main trunk until the very end. The main trunk could be identified based on its larger caliber if a neurolysis and fascicle dissection is performed prior to electrode implantation. In order to verify whether fascicles can be identified based on size and position, we next aimed at describing the intraneural topography of the median trunk. For that, we proceeded by sectioning a harvested median nerve embedded in paraffin. The nerve was divided into 11 portions or blocks, each of them subdivided in 3–6 subportions or segments, as shown in Figure 5. The fascicles were labeled and measured according to several morphological parameters (Figure 6). The epineurium area was variable from block to block (mean of 8.41 ± 1.01 mm^2^, *n* = 11 blocks). The differences were more marked, however, in the first block (15.44 ± 0.51 mm^2^, *n* = 3 segments) and those from the upper arm (IX and X, 11.05 ± 0.34 and 13.07 ± 1.19 mm^2^, *n* = 3 segments each, respectively). The eccentricity of the nerve section was similar in all the blocks (mean of 0.65 ± 0.02, *n* = 11 blocks). The endoneurium area, i.e., the total area occupied by the fascicles, showed similar variability (mean of 3.12 ± 0.24, *n* = 11 blocks). We next calculated the ratio between the endoneurium and epineurium area, which indicates the compactness of the nerve and gives an estimate of the relative amount of connective tissue of the section. Here the average value was 0.42 ± 0.03, being lowest in blocks I and II (0.24 ± 0.01 and 0.31 ± 0.01, respectively) as well as blocks IX and X (0.34 ± 0.04 and 0.35 ± 0.03), and highest in block XI (0.68 ± 0.03). These variations probably corresponded to the looser fascicular organization at the regions before exiting branches. Importantly, a strong difference was evident when the different blocks were grouped into regions. Thus, the upper arm blocks presented a significantly higher endoneurium area (4.13 ± 0.23 mm^2^) compared to the forearm blocks (2.46 ± 0.10 and 2.73 ± 0.21 mm^2^ for distal and proximal, respectively, *p* < 0.01). This would indicate a higher axonal content in more proximal regions, such as the upper arm. The epineurium area decreased as the nerve traveled distally, from 10.40 ± 1.75 mm^2^ in the upper arm blocks to 7.13 ± 0.59 mm^2^ in the proximal forearm blocks and finally 5.80 ± 0.10 mm^2^ in the distal forearm blocks. The ratio between endo- and epineurium as well as the eccentricity of the epineurium remained unchanged (data not shown).

**Figure 7:**
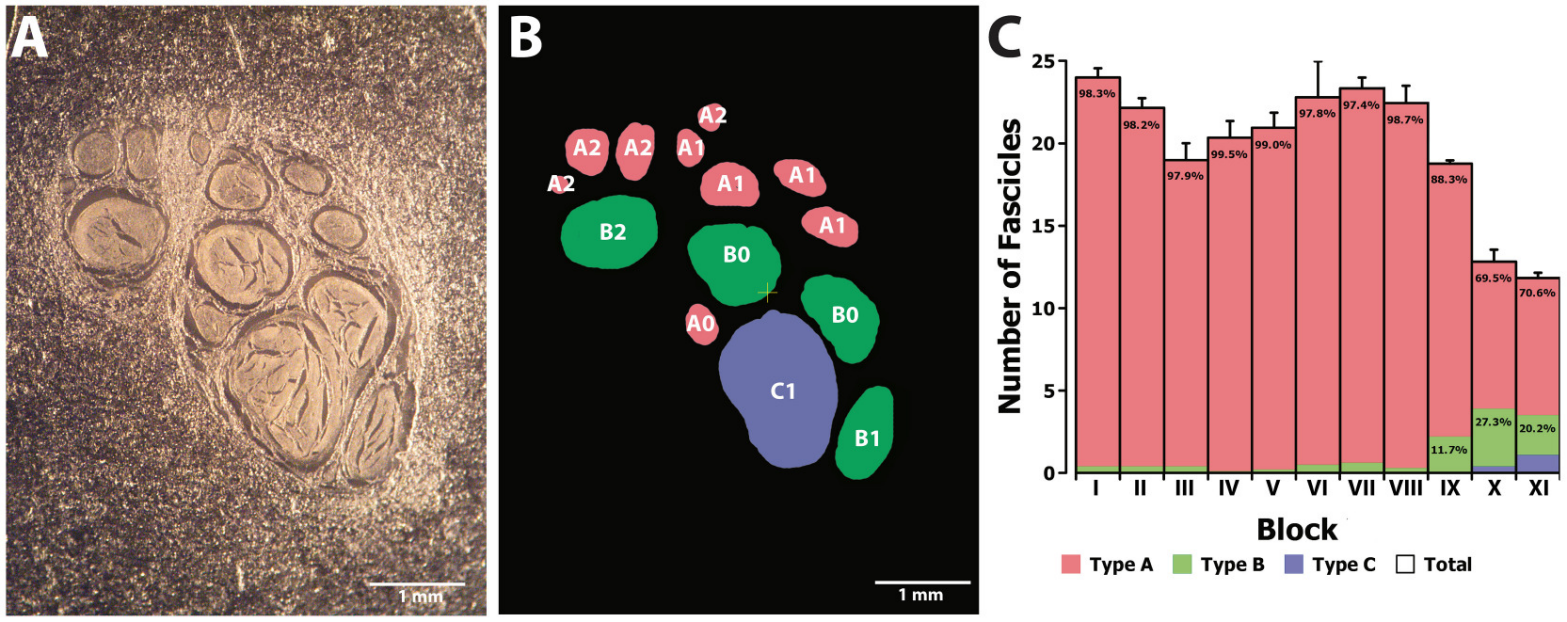
**Clustering analysis of fascicles along the median nerve**. **(A)** Example of a non-stained slice embedded in paraffine corresponding to one of the proximal segments of the median nerve. **(B)** Color map of the slice in **(A)** indicating the membership of each of the fascicles to the clusters corresponding to size (A, B, and C; red, green, and blue, respectively) and distance to the nerve axis (0, 1, and 2). **(C)** Average number of fascicles for each of the blocks and proportion of fascicles belonging to each of the size clusters (A, B, and C; red, green, and blue, respectively).

**Figure 8:**
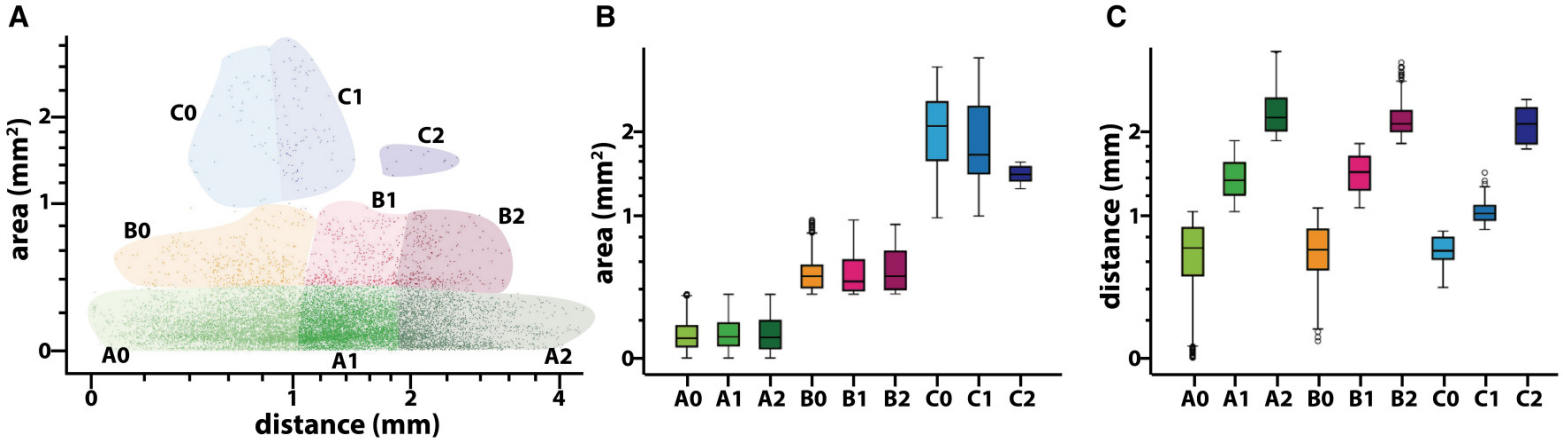
**Results of the clustering analysis for the fascicles composing a median nerve**. **(A)** Scatter plot of the values of area (in mm^2^ and distance to the nerve axis (in mm) for each of the fascicles (*n* = 18,122). Each of the six different colors corresponds to the combinations of membership to the clusters for size (A, B, and C; red, green, and blue, respectively) and distance (0, 1, and 2). Both axis are in a logarithmic scale. **(B,C)** Box-plot showing the statistical values for area and distance to the nerve axis for each of the six different fascicle clusters. Y-axis is in a logarithmic scale.

Next we aimed at analyzing the three dimensional organization of the segments so we could understand how fascicles travel inside the nerve. For that, a 3D reconstruction of the slices was generated using a computer graphics software (Houdini 13.0.665, SideFX, USA). The procedure was iteratively repeated until the whole geometry of the segment could be generated (Figures S2–S12). The model of the nerve could be thus manipulated in 3D space for its examination. A planar lateral projection and an average projection of the 3D stack along the nerve axis were made for examining the changes in the position of the fascicles (Figure 6). As described earlier, the position of the fascicles was found to remain very constant along the length of the segment. On average, we found 19.85 ± 1.23 fascicles per section. Interestingly, the number of fascicles was significantly different from the forearm and the upper arm. Whereas in the forearm, there was an average of 21.87 ± 0.58 fascicles, only 12.81 ± 0.73 and 11.81 ± 0.32 were found in blocks X and XI, respectively (*p* < 0.001). Next we used a cluster analysis to characterize the fascicles according to two features: size of the fascicle (i.e., the area) and the distance to the centroid of the slice (i.e., nerve axis). Using a *k*-means procedure, the fascicles could be satisfactorily classified into six groups: three representing the size differences (group A, B, and C), and other three representing the distance differences (group 0, 1, and 2) (Figure 7). The values for each of the groups are summarized in Table 1. The results showed that most of the fascicles belonged to the group of smallest size (group A: 94%). In more proximal segments, larger fascicles appeared. Fascicles belonging to group B were rare in the forearm and more abundant in the upper arm blocks (0.36 ± 0.03 fascicles, 1.6%, and 2.70 ± 0.40 fascicles, 18.7%). The largest fascicles, in group C, were exceptional (0.8%) and appeared only at the upper arm blocks (0.5 ± 0.3 fascicles, 3.4%). Interestingly, fascicles were homogeneously distributed along the nerve section despite the size (Figure 8).

**Table 1:** **Values for area and distance to the nerve axis for each of the six different fascicle clusters**.

	***N***	**Area (mm^2^)**	**Distance (mm)**
A0	6200 (34.20%)	0.12	0.68
A1	7863 (43.40%)	0.13	1.40
A2	2948 (16.30%)	0.13	2.35
B0	330 (1.80%)	0.52	0.68
B1	341 (1.90%)	0.52	1.46
B2	288 (1.60%)	0.55	2.21
C0	42 (0.20%)	2.02	0.68
C1	98 (0.50%)	1.90	1.05
C2	12 (0.10%)	1.45	2.11
Total	18122 (100.00%)	0.16	1.31

These results show that fascicles cannot be characterized solely based on their size or position within the nerve trunk after the epicondylar landmark.

### 3.3. Motor axons are heterogeneously distributed in proximal median nerve segments

**Figure 9:**
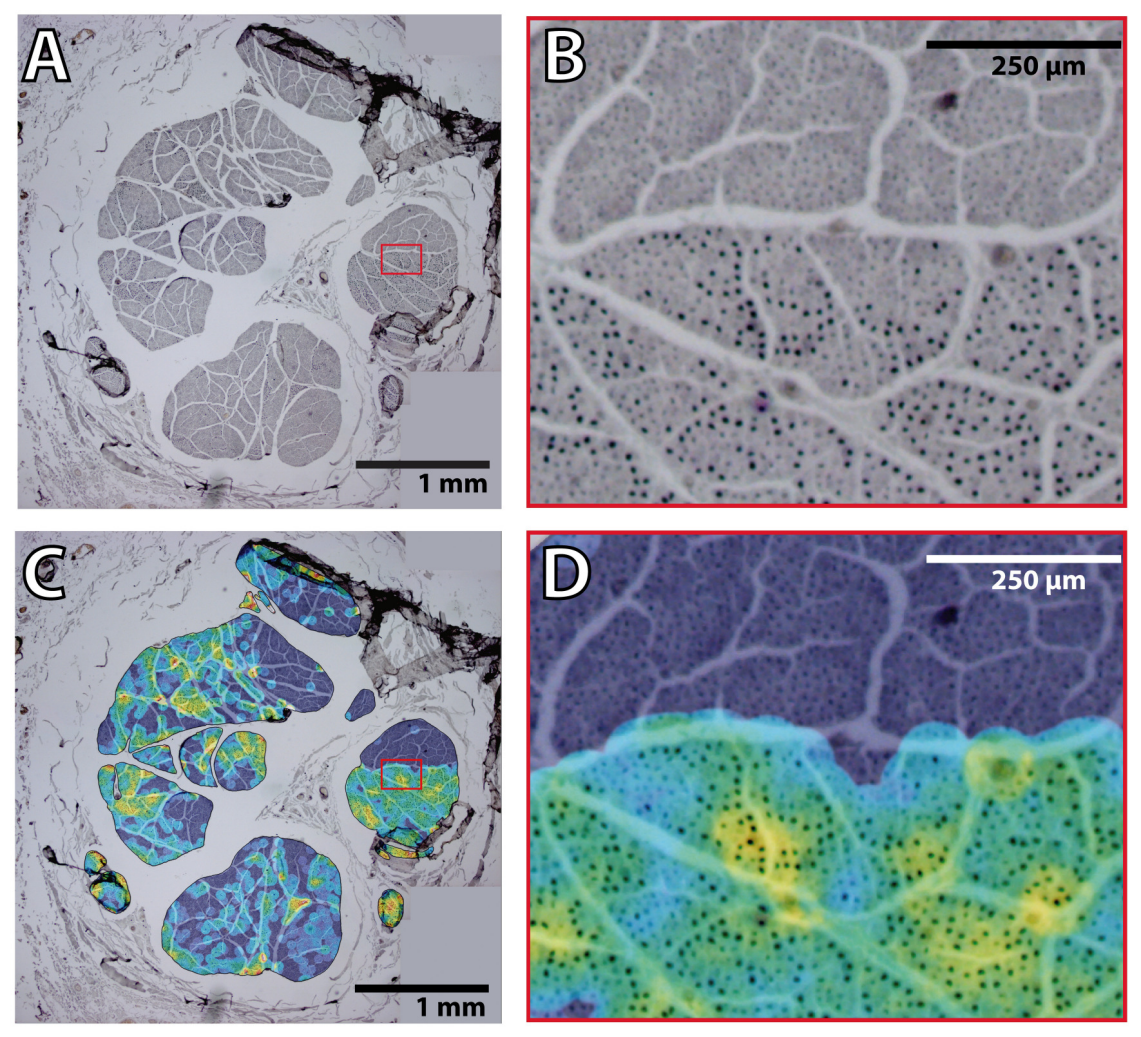
**Heterogeneous distribution of motor fibers in the proximal median nerve**. **(A)** Example of a merge of four pictures obtained at 4 × magnification of the ChAT staining in the median nerve. Red box indicates the region of interest for **(B)**. **(B)** Region indicated in the red box of **(A)** showing the hetereneous pattern of ChAT^+^ axon fibers. **(C)** Overlayed color map on top of the same image as in **(A)** showing the average ratio between ChAT^+^ and total number of axons in an area of radius 45 pixels (corresponding in this case to 3–5 axons). Color map shows in black/blue the lowest values and in red/white the highest ones. Red box indicates the region of interest for **(D)**. **(D)** Region indicated in the red box of **(C)** showing the hetereneous pattern of ChAT^+^ axon fibers with the overlayed ratio map as in **(C)**.

Our results indicated that motor fascicles branch out the main trunk early, in periepicondylar regions, and then they travel in the main trunk for a short distance, occupying a constant location in the nerve section until the corresponding exit point is reached. Proximal to the epicondyle landmark, fascicle size differences allow for visual identification. It is unknown whether the fascicle caliber correlates with the type of information (sensory or motor) carried by the fascicle. To answer the question whether the fascicles in the supraepicondylar region of the median nerve can be discriminated according to the information content, we performed immunohistochemical labeling of motor axons using ChAT at two levels of this part of the nerve (at nFD = 120%, *n* = 3, and nFD = 140%, *n* = 2). The total number of axons were similar in the proximal and distal segments (20,428 ± 1249, *n* = 3, and 20,028 ± 2627, *n* = 2, respectively). The number of ChAT^+^ axons was also similar for both segments (2836 ± 414, *n* = 3, and 2333 ± 179, *n* = 2, respectively), which indicated that approximately one eighth of the axons at those levels targeted muscle fibers. Exceptionally, purely motor fascicles (only one in one unique specimen in both levels) or sensory fascicles (only one in one specimen in distal level) were identified. Fascicles with a motor axonal density over 2 times the standard error of the mean appeared to be located in boundary areas of the nerve, but no pattern could be determined due to the variability between the samples. When we analyzed the motor axon location, we observed that ChAT^+^ axons were heterogeneously distributed in the fascicles (Figure 9). Rather than being randomly mingled with the rest of axons, motor axons were concentrated in patches at certain areas. The location of these highly dense motor patches seemed to be, however, not dependent on size or position of the fascicle.

This result seems to indicate that a certain degree of functional selectivity could be achieved if a neural interface is inserted close to one of those highly dense motor patches. However, identification of these areas is *a priori* not possible during a electrode implantation procedure.

## 4. Discussion

This study provides a topographical description of the human median nerve for its application in neuroprosthetic surgery. Currently, the implantation procedure for a neural interface in patients is not standardized. Commonly, the surgeon improvises the intervention based on previous experience in nerve repair, microsurgical techniques, or similar procedures. From the studies performed in animal models, particularly the rat sciatic nerve (Badia et al., 2010), that have characterized different types of neural interfaces, several conclusions could be extrapolated, which together with our results might help in the planning of the implantation procedure. Indeed, a detailed description of the internal topography of the nerve is an essential decision-making knowledge for the planification of neuroprosthetic surgeries.

The distribution of the fascicles inside the median nerve, especially in the main trunk, has been a subject of debate for years. Sunderland (1945) showed that there was no consistent fascicular pattern for the main arm nerves. From our statistical analysis of the median nerve, we observe that the motor fibers traveling in the upper arm segment organize themselves within the proximal portion of the forearm (about 50 mm from the epicondyles) into the muscular branches responsible for wrist and hand flexion. From the perspective of neuroprosthetic surgery, we can then divide the median nerve based on our description into three main regions: the proximal segment, where the median nerve travels in the upper arm along the humerus bone; the early distal segment, anatomically correlated with the antecubital fossa; and the late distal segment, from the origin point of the interosseous nerve to the entry of the median nerve in the hand through the carpal tunnel. The proximal segment corresponds to a region where, as demonstrated by ChAT^+^ immunostaining, motor axons are heterogeneously distributed and no prediction of their destination can be established *a priori*. In the early distal segments, the topography of the median trunk starts to be defined and fascicles can be identified to a certain probability by its distance to the epicondylar landmark and location within the nerve bundle. This short segment seems to be the optimal for neuroprosthetic applications since, as we show, all the motor fibers for hand function are present and grouped in fascicles. Noticeably, the nerve fibers of the muscles that generate control signals for myoelectric prostheses (Ison and Artemiadis, 2014) exit the main trunk precisely at this level. The distal segment contains small fascicles, which provide innervation to the intrinsic muscles of the hand. Our description shows that no major muscular groups of the forearm receive innervation from this part of the nerve. It is then presumed that these fascicles contain fibers coming from the hand touch receptors and are a suitable target for closed-loop prostheses (Raspopovic et al., 2014).

It is considered that a higher selectivity implies a greater invasiveness (Navarro et al., 2005). From all the neural interfaces, cuff electrodes are considered the least invasive since they are placed around the nerve without penetrating it. Conversely, they have a low signal-to-noise ratio (SNR) because the neural signals must travel from the inner of the nerve to the outer epineural surface where the active contacts are located. The geometry of the nerve makes it difficult to access the inner signal sources and make fascicle discrimination more computationally intensive and complicated (Zariffa et al., 2011). Based on this, cuff electrodes are more suitable for thin nerves, where signal degradation due to distance is minimal. From our description, the distal region of the median nerve, in the forearm, seems to be the most appropriate location for these kind of electrodes. Here the nerve is organized in several individual small fascicles aiming at well defined targets, mostly responsible for selective hand and wrist movements, which eases the signal processing. In a hypothetical scenario, the surgeon might perform a careful microdissection to expose these bundles and implant a microcuff electrode around each one of them.

A particularly interesting design of cuff electrode is the flat interface nerve electrode (FINE). This electrode forces a reshaping of the nerve so the bundles lie flat (Caparso et al., 2008). Although commonly flatten nerve as the femoral (Schiefer et al., 2010) are the main targets, FINEs have been implanted in the median nerve in the context of hand prostheses (Tan et al., 2014). Our study shows that the ratio between the epineurium and endoneurium is particularly high in distal forearm regions and that here size differences between the fascicles are minimal. This would imply that an orderly translocation of fascicles could be effectively achieved without compression or damage. The resulting advantage that this would offer is that, in the case of FINE implantation in the distal part of the median nerve, fascicle exposure by microsurgical neurolysis might not be needed.

Intrafascicular electrodes (such as LIFE and TIME) are invasive electrodes, which are implanted along or across the nerve, providing a high selectivity without significant damage (Lago et al., 2007; Badia et al., 2011). These electrodes seem to be especially suitable in situations where a certain degree of segregation is present among the fascicles, even though they are not physically separated. Accordingly, the region where the major motor branches are expected to exit, i.e., the 95% confidence interval or the average branching point of the interosseous nerve, appears to be the best location for intrafascicular electrodes. Our observations show that here the fibers targeting the lower arm muscles are well organized in fascicles. However, the presence of axonal collaterals and anatomosis among fascicles makes dissection risky. Furthermore, we show that the size relation between the main trunk and the rest of the branches starts to be noticeable here, and therefore shape remodeling of the nerve by a FINE might not be optimal. Intrafascicular electrode appears to be, however, ideal for these situations: the flexible shaft can be easily inserted through a small epineural window aiming at one or more fascicles to gain fascicle selectivity.

The USEA is a neural interface with high selectivity and good quality of signal recording. However, the risk of damaging the nerve after implantation is also higher. This electrode is a modification of the microarray electrodes used in the brain cortex (Branner et al., 2001). However, opposite to this location, the nerve is not enclosed in a fixed compartment but attached to mobile structures such muscles, bones, and joints. This constantly creates a relative movement between the rigid electrode shafts and the soft nerve tissue, which with time may produce damage. For this reason, the implantation of USEA in the nerve must assure that the electrode is properly fixed. According to our model, the proximal part of the median nerve appears to be the best location. Here the median nerve travels alongside the humerus bone, so its movement is minimal. In this portion, most of the nerve space is occupied by a main trunk, where fascicle organization is still undefined, but big enough to accommodate the implant. Additionally, the axonal density is high, meaning that the chances for an axon to be close enough to an electrode shaft are high. Therefore, if an USEA is implanted in the main trunk after neurolysis and dissection and fixed in such way that the relative movement between the electrode and the nerve is minimal, a good and precise communication with the nerve could be achieved, as shown in similar experiments in non-human primates (Ledbetter et al., 2013).

## Author contributions

Nerve dissections were performed by JB, AP, AR, and XN. Histological sectioning and processing was done by JB and AP. Morphological quantification was carried out by ID, JB, and AP. 3D reconstruction, data analysis, script programming, and statistics were done by ID. Manuscript was written by ID and revised and corrected by JB, AP, AR, and XN. Study design was made by XN.

### Conflict of interest statement

The authors declare that the research was conducted in the absence of any commercial or financial relationships that could be construed as a potential conflict of interest.

## Supplemental data

### Calculation of the branching probability

To describe the branching pattern of a median nerve of at the forearm segment of length *l*, we first counted the number of branches, *B*, that exited at a certain distance, *i*, from beginning of the nerve at the styloid landmark and that innervate a certain muscle, *M*:

(1) $$\begin{array}{l} B_{M}\left(i\right)=0,1,\ldots ;i\in \left[0,l\right] \end{array}$$

In this study, we used *l* = 100% of the nFD to indicate the full length of the forearm. The total number of branches from the median nerve that innervates a muscle *M* can be then calculated as:

(2) $$\begin{array}{l} \overset{l}{\underset{i=0}{\sum }}B_{M}\left(i\right) \end{array}$$

For inter-subject comparison of the branching pattern of the median nerve, we next defined the branching probability, *P*, for a muscle, *M*, at a distance *x* from the beginning of the nerve as the probability of a branch of the median nerve to exit the main trunk within the region between *x* and the total length of the nerve, *L*, so it provides innervation to the muscle:

(3) $$\begin{array}{l} P_{M}\left(x\right)=\frac{\overset{L}{\underset{i=x}{\sum }}B_{M}\left(i\right)}{\overset{l}{\underset{i=0}{\sum }}B_{M}\left(i\right)};x\in \left[0,L\right] \end{array}$$

For our calculations, *L* represents the full length of the median nerve, from the styloid landmark to the brachial plexus, and, therefore, it implies that *l* ≤ *L*. This is possible since due to the high variability in nerve branching, some of the branches for a muscle may originate proximal to the epicondyle landmark (*nFD* ≥ 100%)

Figure S1 **Schematics showing the origin and destination of five different median nerves with the distances from the styloid landmark to the exit point next to it in cm**. The gray area indicates the region between the epicondylar landmark and the styloid one used for normalization of distances (normalized forearm distance, nFD). The vertical axis on the left shows the values of the nFD in percentage. Abbreviations in text.

Figure S2 **Reconstruction of each of the segments of the median nerve corresponding to block I**. Each segment is represented in one independent panel. Segments are grouped into blocks. For each of the panels, the three elements represent (left) the lateral planar projection of the fascicle fibers along the nerve (vertical scale = 250 μm, horizontal scale = 1 mm); (top) probability distribution of the fascicles along the nerve using a color jet map gradient (black/blue corresponds to lowest values and red/white to highest ones; scale = 1 mm); (bottom) frequency distribution of the fascicles grouped according the clustering parameters of size (*A*,*B*, and *C*; red, green, and blue, respectively) and distance to the nerve axis (0, 1, 2).

Figure S3 **Reconstruction of each of the segments of the median nerve corresponding to block II**. See caption for Figure S2.

Figure S4 **Reconstruction of each of the segments of the median nerve corresponding to block III**. See caption for Figure S2.

Figure S5 **Reconstruction of each of the segments of the median nerve corresponding to block IV**. See caption for Figure S2.

Figure S6 **Reconstruction of each of the segments of the median nerve corresponding to block V**. See caption for Figure S2.

Figure S7 **Reconstruction of each of the segments of the median nerve corresponding to block VI**. See caption for Figure S2.

Figure S8 **Reconstruction of each of the segments of the median nerve corresponding to block VII**. See caption for Figure S2.

Figure S9 **Reconstruction of each of the segments of the median nerve corresponding to block VIII**. See caption for Figure S2.

Figure S10 **Reconstruction of each of the segments of the median nerve corresponding to block IX**. See caption for Figure S2.

Figure S11 **Reconstruction of each of the segments of the median nerve corresponding to block X**. See caption for Figure S2.

Figure S12 **Reconstruction of each of the segments of the median nerve corresponding to block XI**. See caption for Figure S2.

Table S1 **Summary of the anatomical values**.

## Abbreviations

ChAT: Choline AcetylTransferase
ET: Epitroclear muscles
FCR: Flexor Carpi Radialis
FDP: Flexor Digitorum Profundus
FDS: Flexor Digitorum Superficialis
FPL: Flexor Pollicis Longus
nFD: normalized Forearm Distance
PL: Palmaris Longus
PQ: Pronator Quadratus
PT: Pronator Teres.

## References

[B1] Badia J. Boretius T. Pascual-Font A. Udina E. Stieglitz T. (2011). Biocompatibility of chronically implanted transverse intrafascicular multichannel electrode (time) in the rat sciatic nerve. IEEE Trans. Biomed. Eng. 58, 2324–2332. 10.1109/TBME.2011.2153850 21571604

[B2] Badia J. Pascual-Font A. Vivó M. Udina E. Navarro X. (2010). Topographical distribution of motor fascicles in the sciatic-tibial nerve of the rat. Muscle Nerve 42, 192–201. 10.1002/mus.21652 20544926

[B3] Barcelo C. Faruch M. Lapegue F. Bayol M.-A. Sans N. (2013). 3-T MRI with diffusion tensor imaging and tractography of the median nerve. Eur. Radiol. 23, 3124–3130. 10.1007/s00330-013-2955-2 23832318

[B4] Belter J. T. Segil J. L. Dollar A. M. Weir R. F. (2013). Mechanical design and performance specifications of anthropomorphic prosthetic hands: a review. J. Rehabil. Res. Dev. 50, 599–617. 10.1682/JRRD.2011.10.0188 24013909

[B5] Bernsen J. (1986). Dynamic thresholding of gray-level images, in Proceeding of the Eight International conference Pattern Recognition. (Paris), 1251–1255.

[B6] Boretius T. Badia J. Pascual-Font A. Schuettler M. Navarro X. Yoshida K.. (2010). A transverse intrafascicular multichannel electrode (time) to interface with the peripheral nerve. Biosens. Bioelectron. 26, 62–69. 10.1016/j.bios.2010.05.010 20627510

[B7] Borton D. Micera S. Millán J. D. R. Courtine G. (2013). Personalized neuroprosthetics. Sci. Transl. Med. 5:210rv2. 10.1126/scitranslmed.3005968 24197737

[B8] Branner A. Stein R. B. Normann R. A. (2001). Selective stimulation of cat sciatic nerve using an array of varying-length microelectrodes. J. Neurophysiol. 85, 1585–1594. Available online at: http://jn.physiology.org/content/85/4/1585.long 11287482 10.1152/jn.2001.85.4.1585

[B9] Caparso A. V. Durand D. M. Mansour J. M. (2008). A nerve cuff electrode for controlled reshaping of nerve geometry. J. Biomater. Appl. 24, 247–273. 10.1177/0885328208097426 18987020 PMC3569731

[B10] Collinger J. L. Wodlinger B. Downey J. E. Wang W. Tyler-Kabara E. C. Weber D. J.. (2013). High-performance neuroprosthetic control by an individual with tetraplegia. Lancet 381, 557–564. 10.1177/0885328208097426 23253623 PMC3641862

[B11] del Valle J. Navarro X. (2013). Interfaces with the peripheral nerve for the control of neuroprostheses. Int. Rev. Neurobiol. 109, 63–83. 10.1016/B978-0-12-420045-6.00002-X 24093606

[B12] Dhillon G. S. Lawrence S. M. Hutchinson D. T. Horch K. W. (2004). Residual function in peripheral nerve stumps of amputees: implications for neural control of artificial limbs. J. Hand Surg. (Am.) 29A, 605–615. 10.1016/j.jhsa.2004.02.006 15249083

[B13] Di Pino G. Denaro L. Vadalà G. Marinozzi A. Tombini M. Ferreri F.. (2014). Invasive neural interfaces: the perspective of the surgeon. J. Surg. Res. 188, 77–87. 10.1016/j.jss.2013.12.014 24433868

[B14] Ison M. Artemiadis P. (2014). The role of muscle synergies in myoelectric control: trends and challenges for simultaneous multifunction control. J. Neural Eng. 11:051001. 10.1088/1741-2560/11/5/051001 25188509

[B15] Lago N. Navarro X. (2006). Correlation between target reinnervation and distribution of motor axons in the injured rat sciatic nerve. J. Neurotrauma 23, 227–240. 10.1089/neu.2006.23.227 16503806

[B16] Lago N. Yoshida K. Koch K. P. Navarro X. (2007). Assessment of biocompatibility of chronically implanted polyimide and platinum intrafascicular electrodes. IEEE Trans. Biomed. Eng. 54, 281–290. 10.1109/TBME.2006.886617 17278585

[B17] Lawrence S. Dhillon G. Jensen W. Yoshida K. Horch K. (2004). Acute peripheral nerve recording characteristics of polymer-based longitudinal intrafascicular electrodes. IEEE Trans. Neural Syst. Rehabil. Eng. 12, 345–348. 10.1109/TNSRE.2004.831491 15473197

[B18] Ledbetter N. M. Ethier C. Oby E. R. Hiatt S. D. Wilder A. M. Ko J. H.. (2013). Intrafascicular stimulation of monkey arm nerves evokes coordinated grasp and sensory responses. J. Neurophysiol. 109, 580–590. 10.1152/jn.00688.2011 23076108 PMC3545468

[B19] Micera S. Rossini P. M. Rigosa J. Citi L. Carpaneto J. Raspopovic S.. (2011). Decoding of grasping information from neural signals recorded using peripheral intrafascicular interfaces. J. NeuroEng. Rehabil. 8:53. 10.1186/1743-0003-8-53 21892926 PMC3177892

[B20] Navarro X. Krueger T. B. Lago N. Micera S. Stieglitz T. Dario P. (2005). A critical review of interfaces with the peripheral nervous system for the control of neuroprostheses and hybrid bionic systems. J. Peripher. Nerv. Syst. 10, 229–258. 10.1111/j.1085-9489.2005.10303.x 16221284

[B21] Pizer S. M. Amburn E. P. Austin J. D. Cromartie R. Geselowitz A. Greer T. (1987). Adaptive histogram equalization and its variations. Comput. Vision Graph. Image Process. 39, 355–368. 10.1016/S0734-189X(87)80186-X

[B22] Planitzer U. Steinke H. Meixensberger J. Bechmann I. Hammer N. Winkler D. (2014). Median nerve fascicular anatomy as a basis for distal neural prostheses. Anat. Anz. 196, 144–149. 10.1016/j.aanat.2013.11.002 24374103

[B23] Raspopovic S. Capogrosso M. Petrini F. M. Bonizzato M. Rigosa J. Di Pino G.. (2014). Restoring natural sensory feedback in real-time bidirectional hand prostheses. Sci. Transl. Med. 6:222ra19. 10.1126/scitranslmed.3006820 24500407

[B24] Raspopovic S. Carpaneto J. Udina E. Navarro X. Micera S. (2010). On the identification of sensory information from mixed nerves by using single-channel cuff electrodes. J. NeuroEng. Rehabil. 7:17. 10.1186/1743-0003-7-17 20423488 PMC2887885

[B25] Rossini P. M. Micera S. Benvenuto A. Carpaneto J. Cavallo G. Citi L.. (2010). Double nerve intraneural interface implant on a human amputee for robotic hand control. Clin. Neurophysiol. 121, 777–783. 10.1016/j.clinph.2010.01.001 20110193

[B26] Sahoo P. K. Soltani S. Wong A. K. C. Chen Y. C. (1988). A survey of thresholding techniques. Comput. Vision Graph. Image Process. 41, 233–260. 10.1016/0734-189X(88)90022-9

[B27] Santello M. Baud-Bovy G. Jörntell H. (2013). Neural bases of hand synergies. Front. Comput. Neurosci. 7:23. 10.3389/fncom.2013.00023 23579545 PMC3619124

[B28] Schiefer M. A. Polasek K. H. Triolo R. J. Pinault G. C. J. Tyler D. J. (2010). Selective stimulation of the human femoral nerve with a flat interface nerve electrode. J. Neural Eng. 7:26006. 10.1088/1741-2560/7/2/026006 20208125 PMC2915830

[B29] Schindelin J. Arganda-Carreras I. Frise E. Kaynig V. Longair M. Pietzsch T.. (2012). Fiji: an open-source platform for biological-image analysis. Nat. Methods 9, 676–682. 10.1038/nmeth.2019 22743772 PMC3855844

[B30] Sezgin M. Sankur B. (2004). Survey over image thresholding techniques and quantitative performance evaluation. J. Electron. Imaging 13, 146–168. 10.1117/1.1631315

[B31] Strollo P. J. Jr. Soose R. J. Maurer J. T. de Vries N. Cornelius J. Froymovich O.. (2014). Upper-airway stimulation for obstructive sleep apnea. N. Engl. J. Med. 370, 139–149. 10.1056/NEJMoa1308659 24401051

[B32] Sun K. Zhang J. Chen T. Chen Z. Chen Z. Li Z.. (2009). Three-dimensional reconstruction and visualization of the median nerve from serial tissue sections. Microsurgery 29, 573–577. 10.1002/micr.20646 19308949

[B33] Sunderland S. (1945). The intraneural topography of the radial, median and ulnar nerves. Brain 68, 243–298. 10.1093/brain/68.4.243 20982793

[B34] Tan D. W. Schiefer M. A. Keith M. W. Anderson J. R. Tyler J. Tyler D. J. (2014). A neural interface provides long-term stable natural touch perception. Sci. Transl. Med. 6:257ra138. 10.1126/scitranslmed.3008669 25298320 PMC5517305

[B35] Yoshida K. Stein R. (1999). Characterization of signals and noise rejection with bipolar longitudinal intrafascicular electrodes. IEEE Trans. Biomed. Eng. 46, 226–234. 10.1109/10.740885 9932344

[B36] Zariffa J. Nagai M. K. Schuettler M. Stieglitz T. Daskalakis Z. J. Popovic M. R. (2011). Use of an experimentally derived leadfield in the peripheral nerve pathway discrimination problem. IEEE Trans. Neural Syst. Rehabil. Eng. 19, 147–156. 10.1109/TNSRE.2010.2091429 21075737

