# Supplementary figures and images for: Fascicular Topography of the Human Median Nerve for Neuroprosthetic Surgery
Source: Front Neurosci. 2016 Jul 1;10:286. doi: 10.3389/fnins.2016.00286 (PMC4929846; doi:10.3389/fnins.2016.00286)

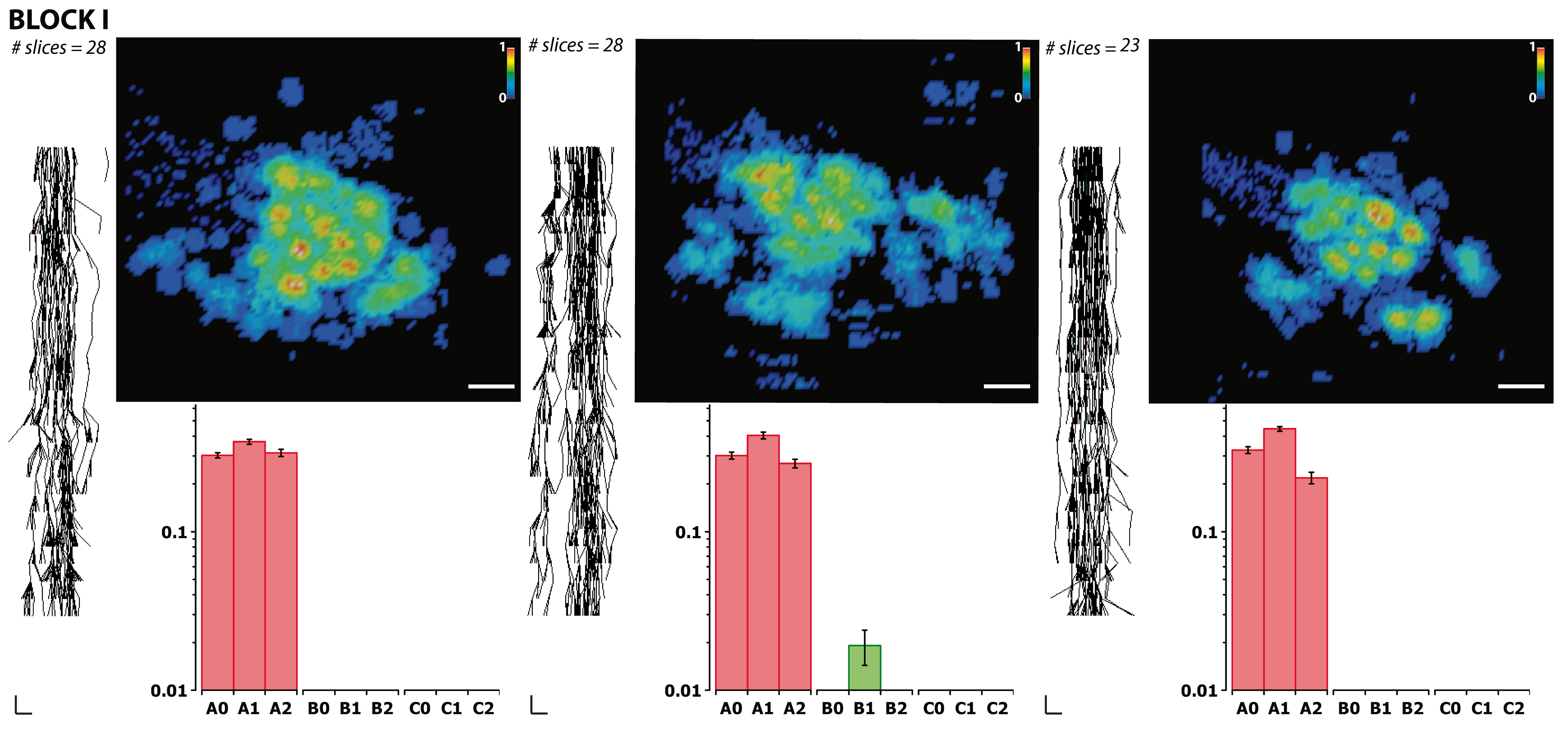

Supplement: Figure S2 — Reconstruction of each of the segments of the median nerve corresponding to block I. Each segment is represented in one independent panel. Segments are grouped into blocks. For each of the panels, the three elements represent (left) the lateral planar projection of the fascicle fibers along the nerve (vertical scale = 250 μm, horizontal scale = 1 mm); (top) probability distribution of the fascicles along the nerve using a color jet map gradient (black/blue corresponds to lowest values and red/white to highest ones; scale = 1 mm); (bottom) frequency distribution of the fascicles grouped according the clustering parameters of size (A,B, and C; red, green, and blue, respectively) and distance to the nerve axis (0, 1, 2). [file Image2.JPEG]

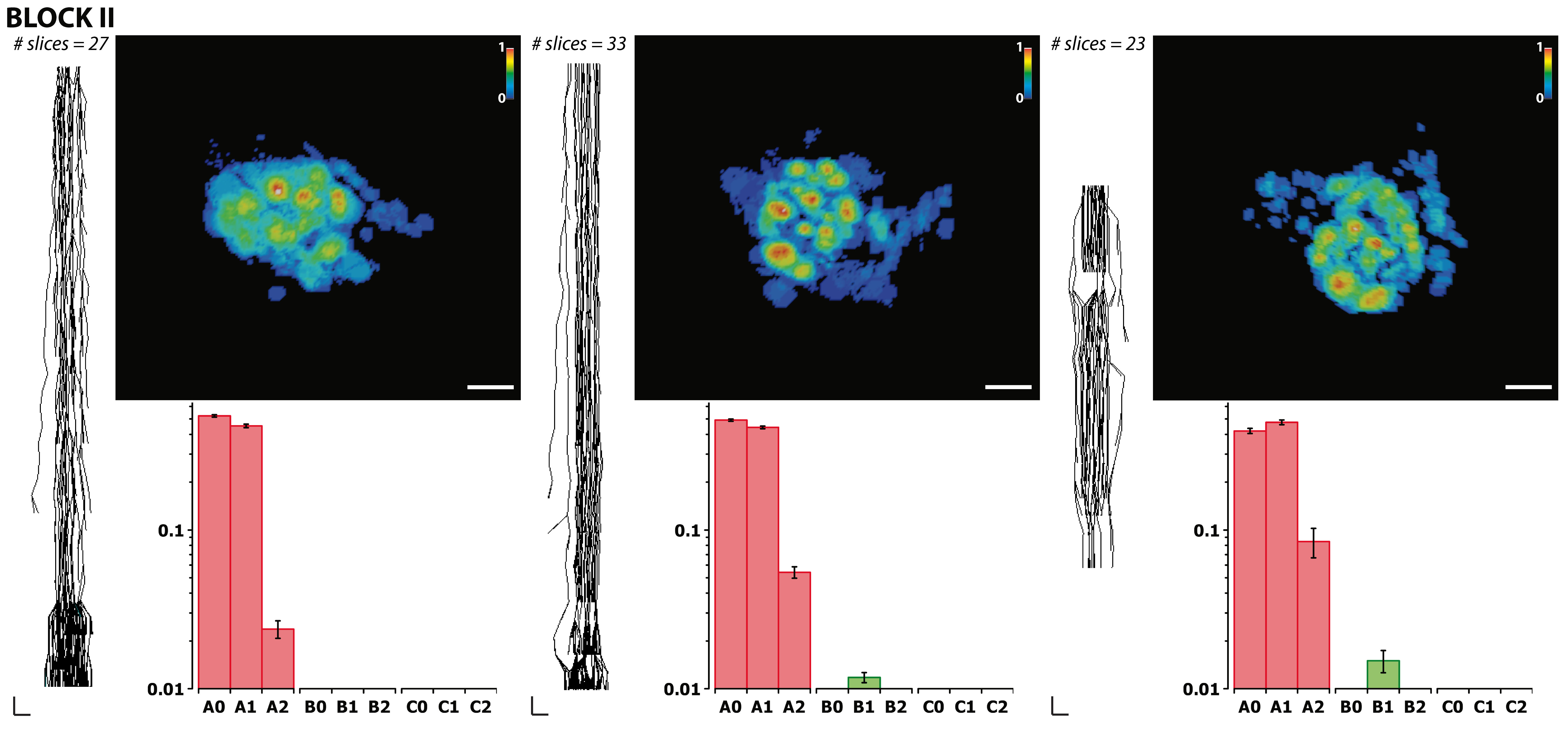

Supplement: Figure S3 — Reconstruction of each of the segments of the median nerve corresponding to block II. See caption for Figure S2. [file Image3.JPEG]

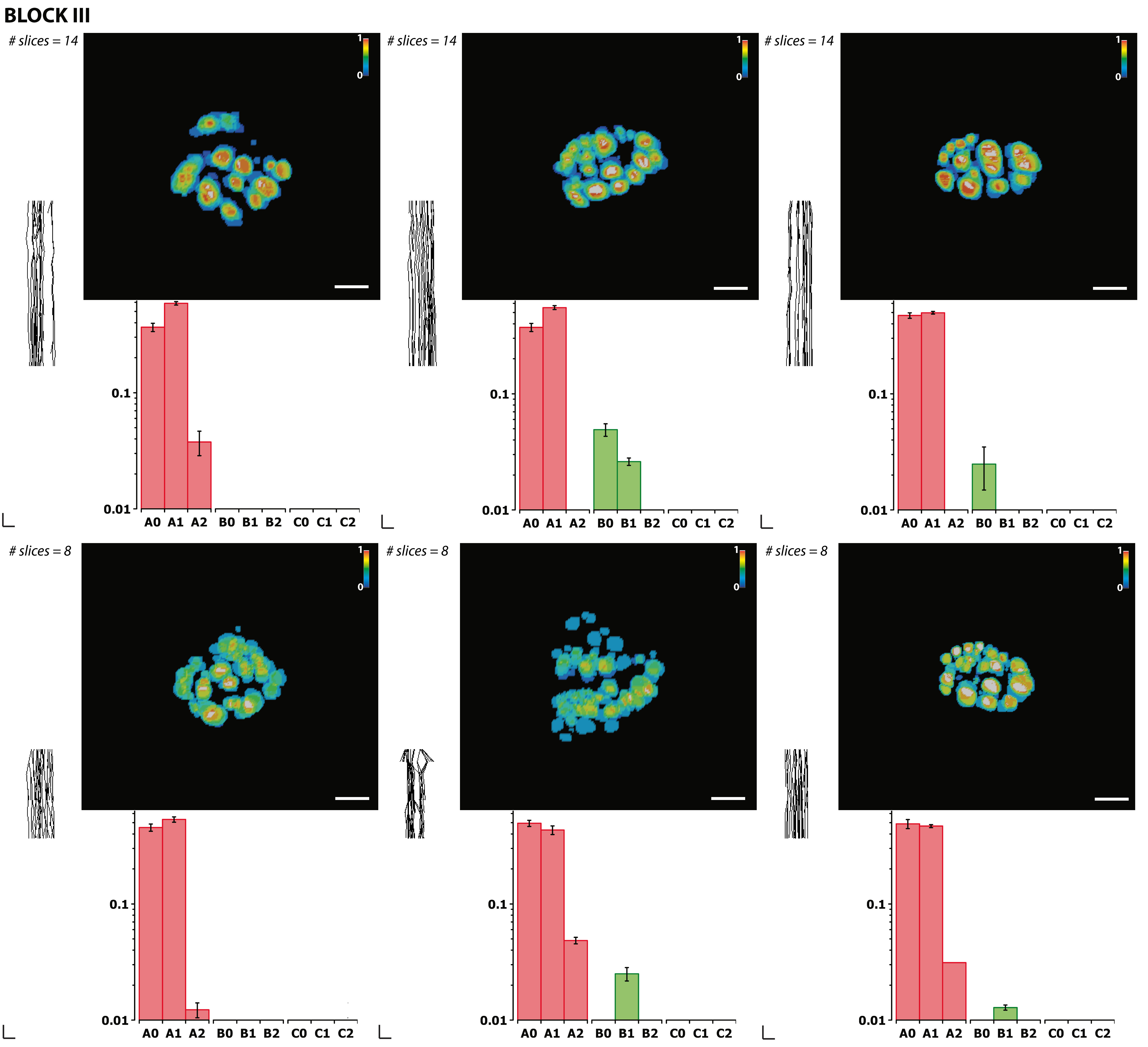

Supplement: Figure S4 — Reconstruction of each of the segments of the median nerve corresponding to block III. See caption for Figure S2. [file Image4.JPEG]

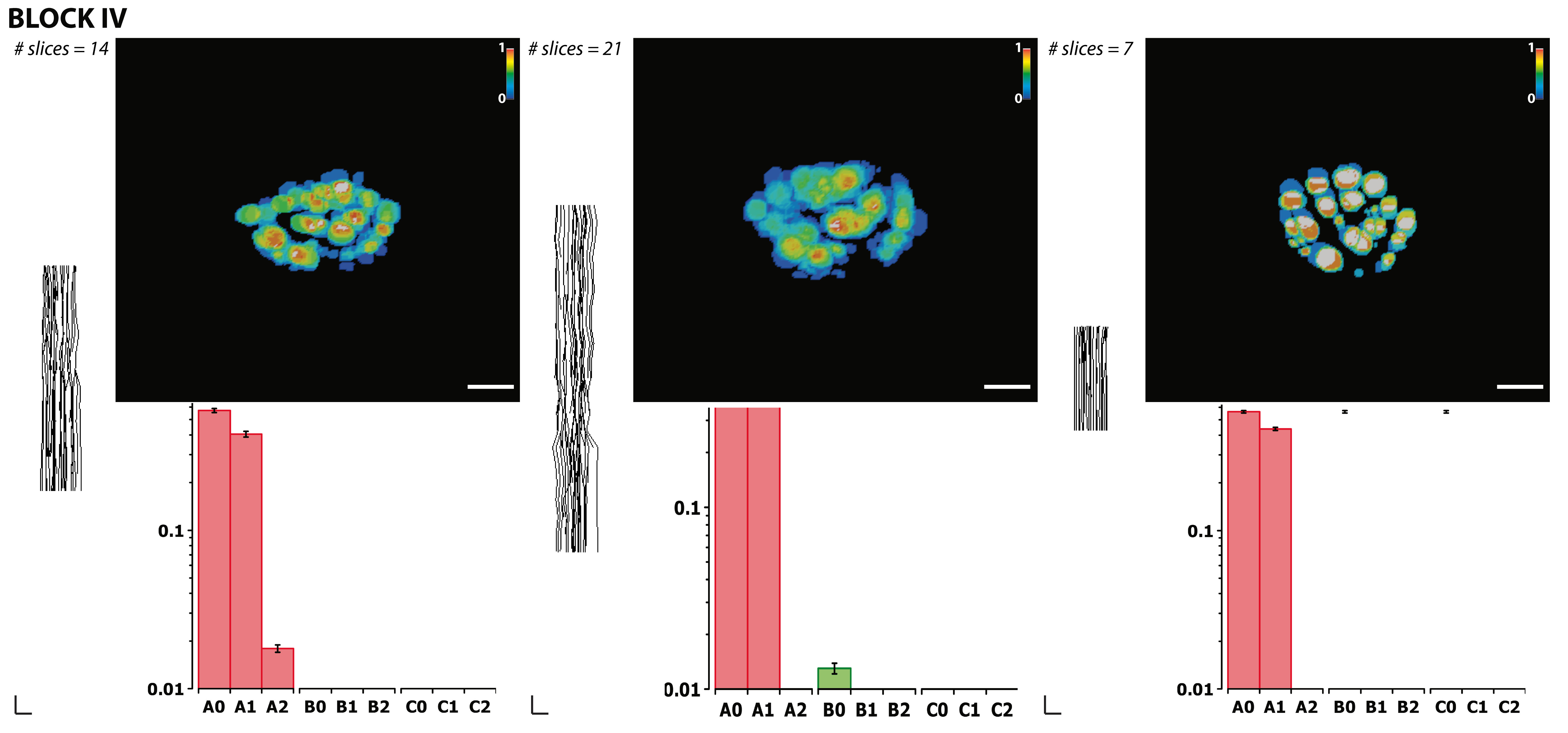

Supplement: Figure S5 — Reconstruction of each of the segments of the median nerve corresponding to block IV. See caption for Figure S2. [file Image5.JPEG]

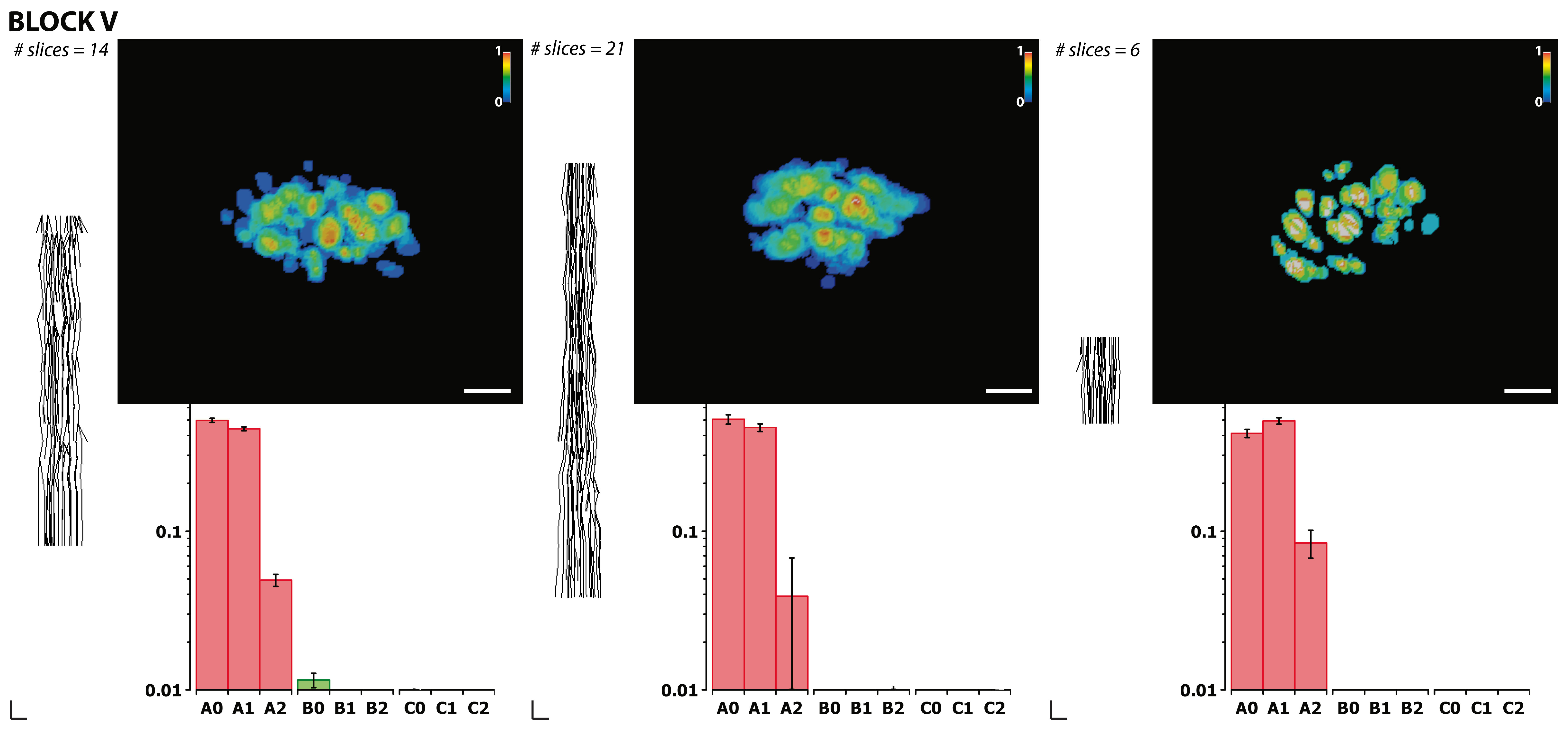

Supplement: Figure S6 — Reconstruction of each of the segments of the median nerve corresponding to block V. See caption for Figure S2. [file Image6.JPEG]

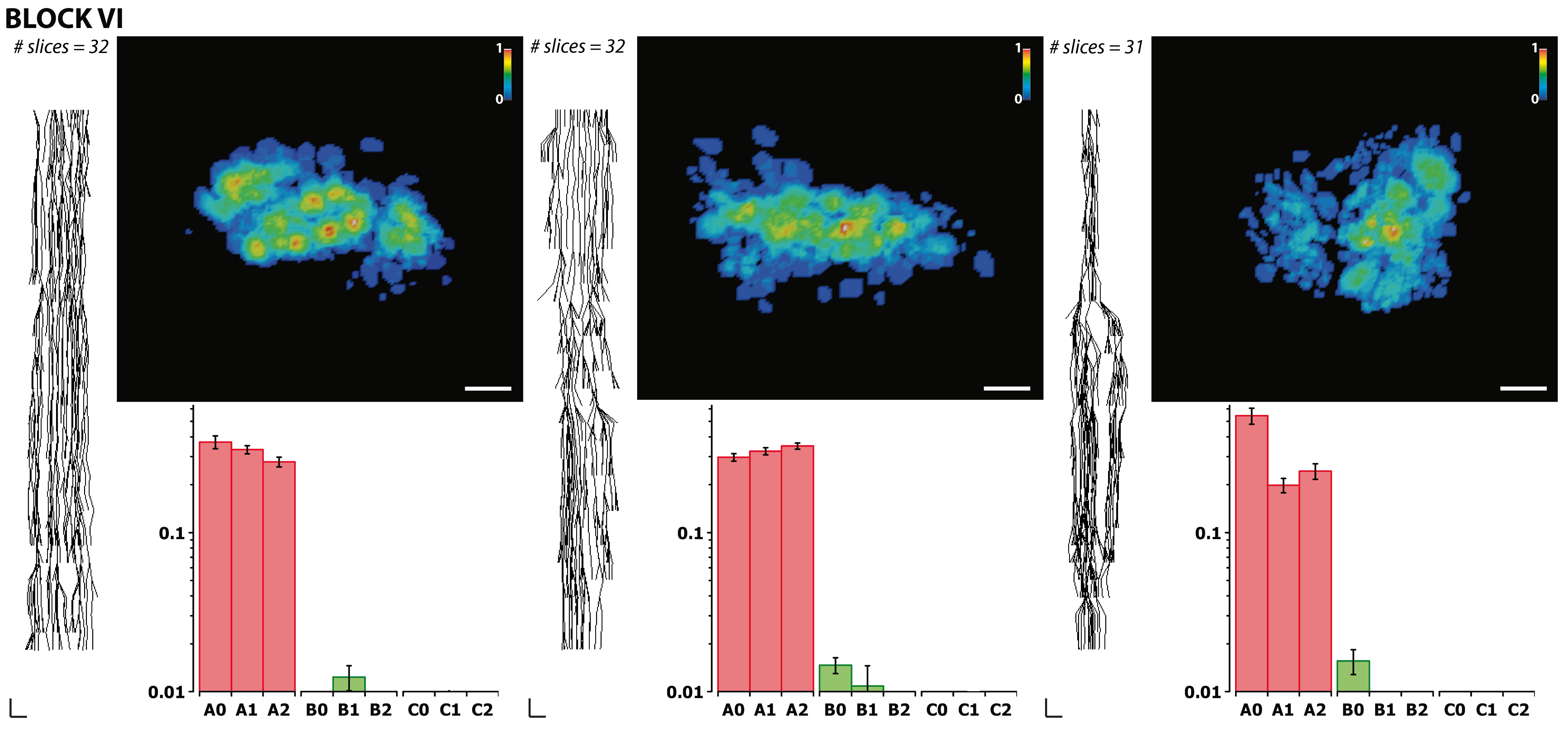

Supplement: Figure S7 — Reconstruction of each of the segments of the median nerve corresponding to block VI. See caption for Figure S2. [file Image7.JPEG]

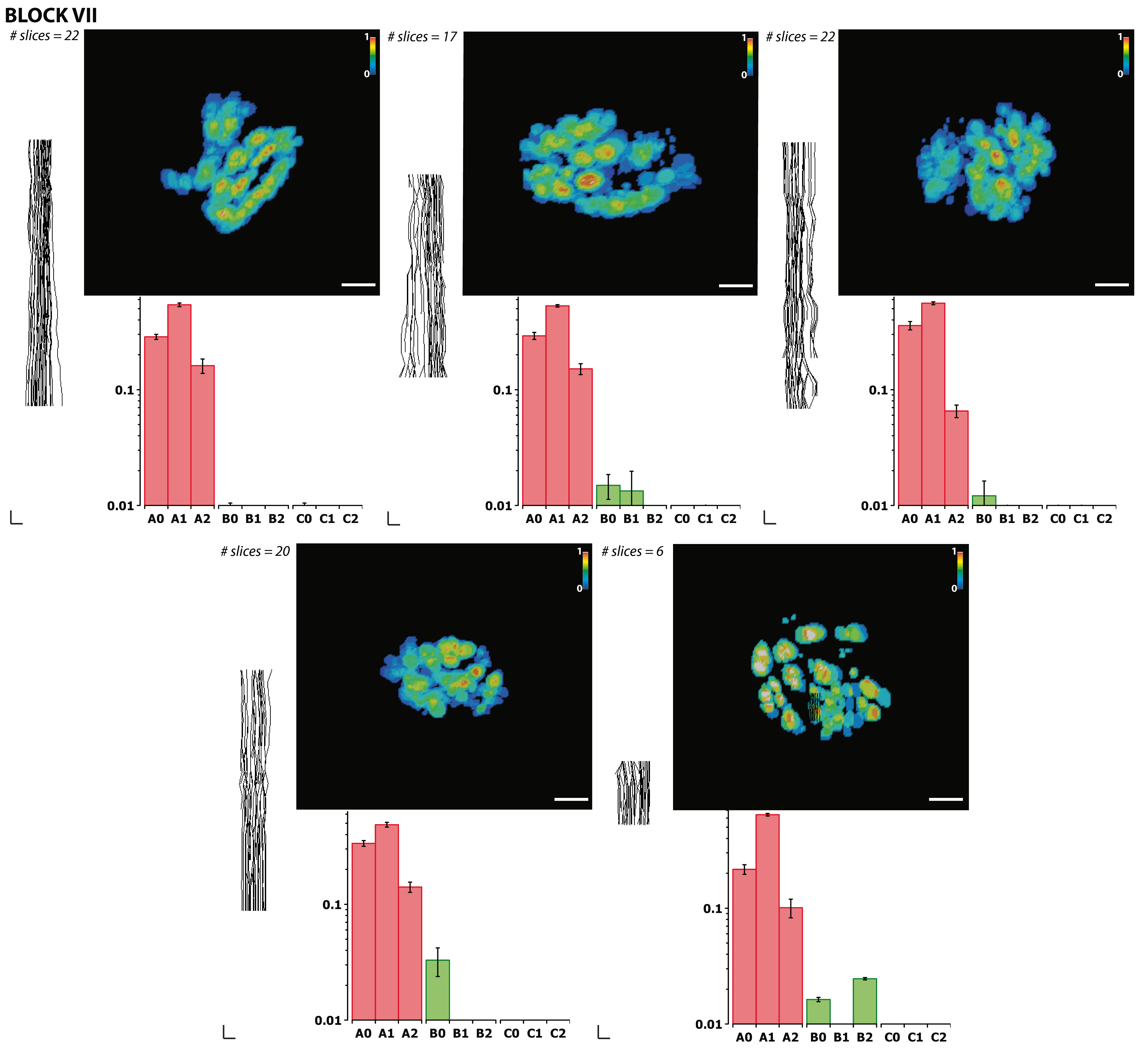

Supplement: Figure S8 — Reconstruction of each of the segments of the median nerve corresponding to block VII. See caption for Figure S2. [file Image8.JPEG]

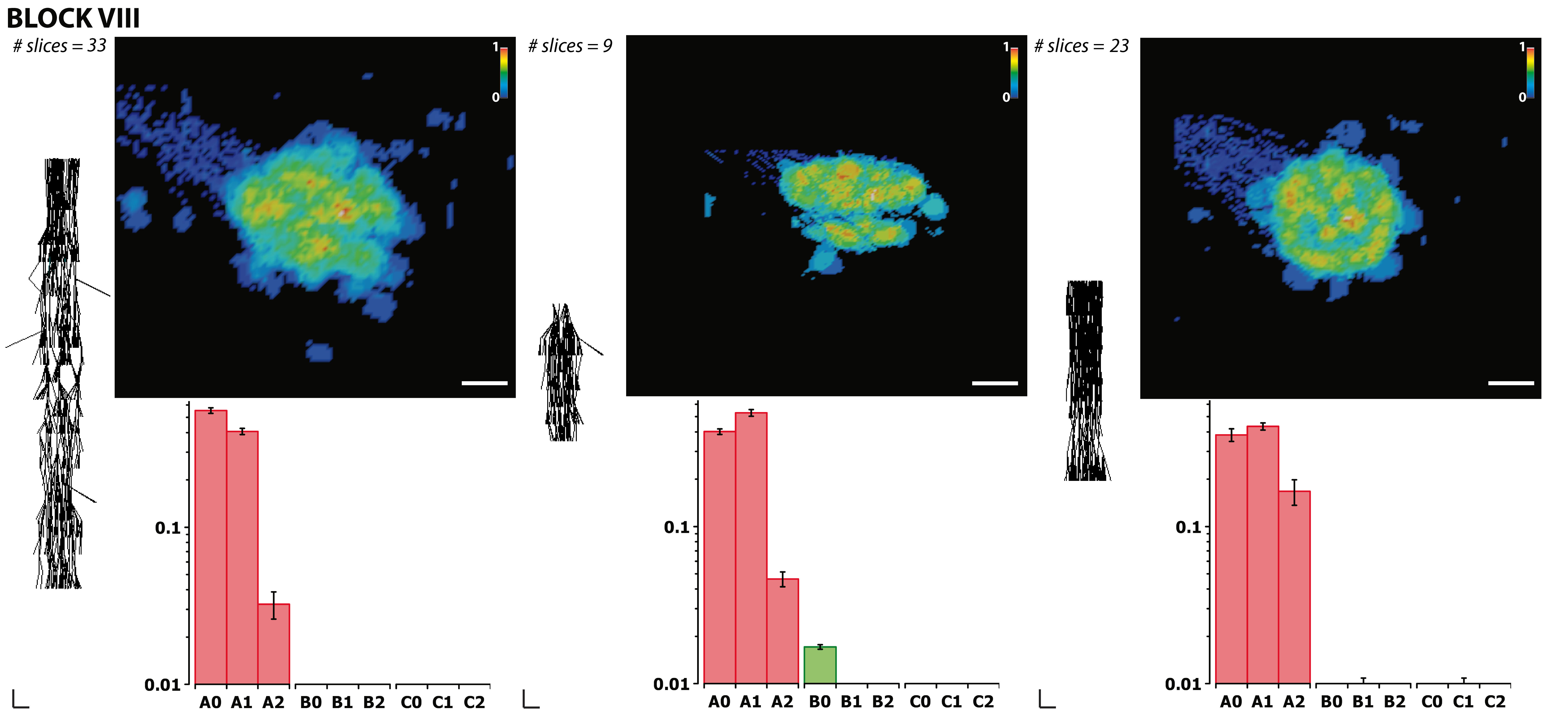

Supplement: Figure S9 — Reconstruction of each of the segments of the median nerve corresponding to block VIII. See caption for Figure S2. [file Image9.JPEG]

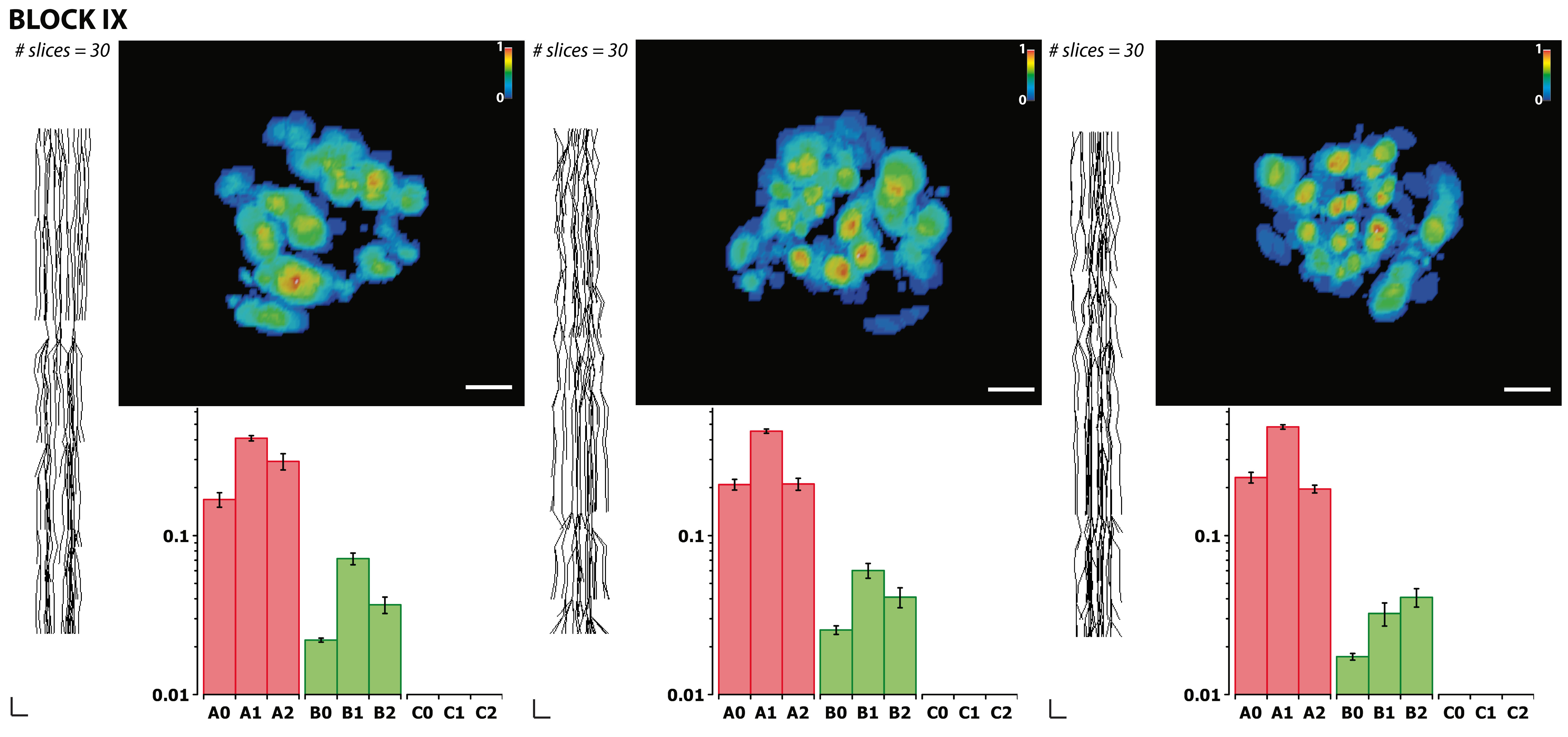

Supplement: Figure S10 — Reconstruction of each of the segments of the median nerve corresponding to block IX. See caption for Figure S2. [file Image10.JPEG]

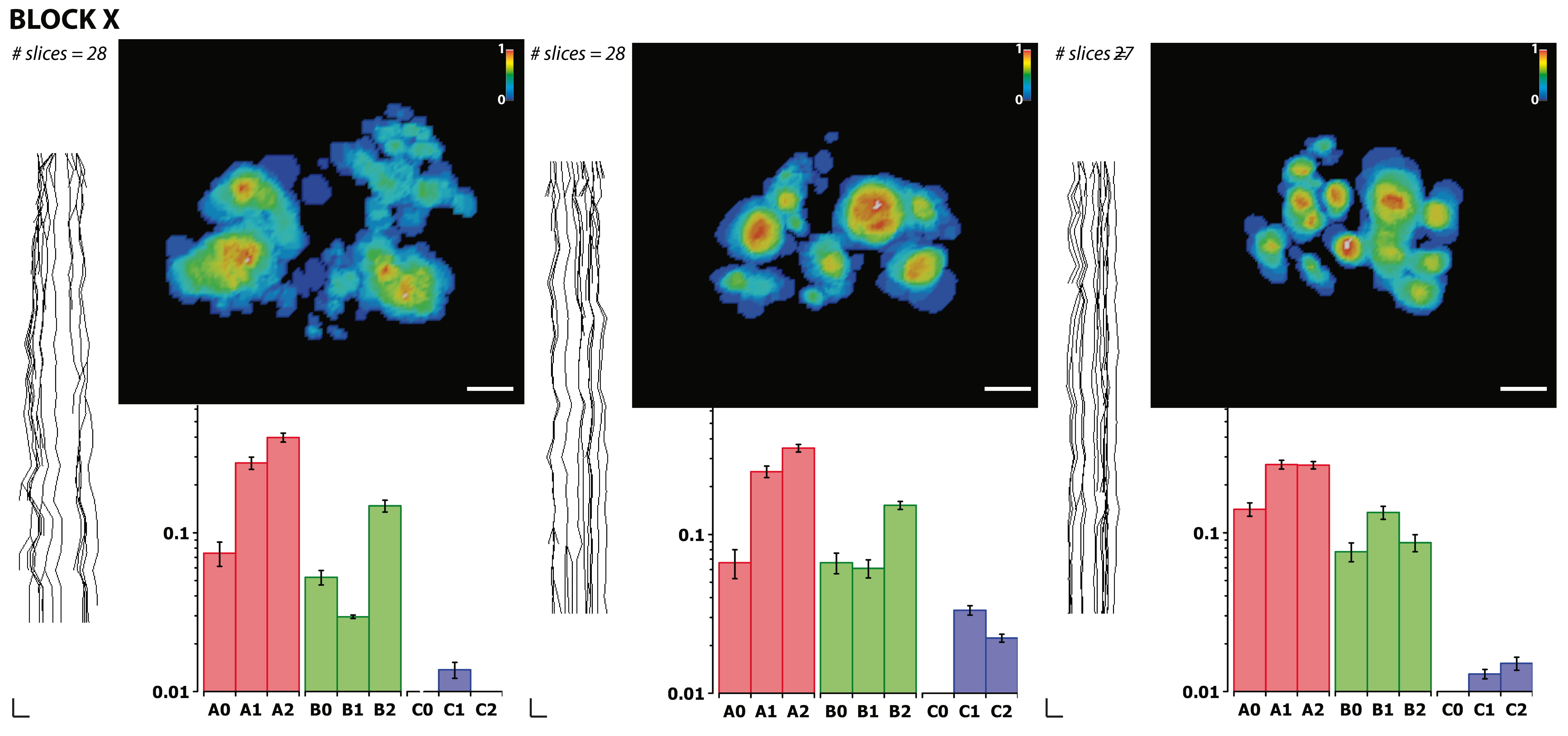

Supplement: Figure S11 — Reconstruction of each of the segments of the median nerve corresponding to block X. See caption for Figure S2. [file Image11.JPEG]

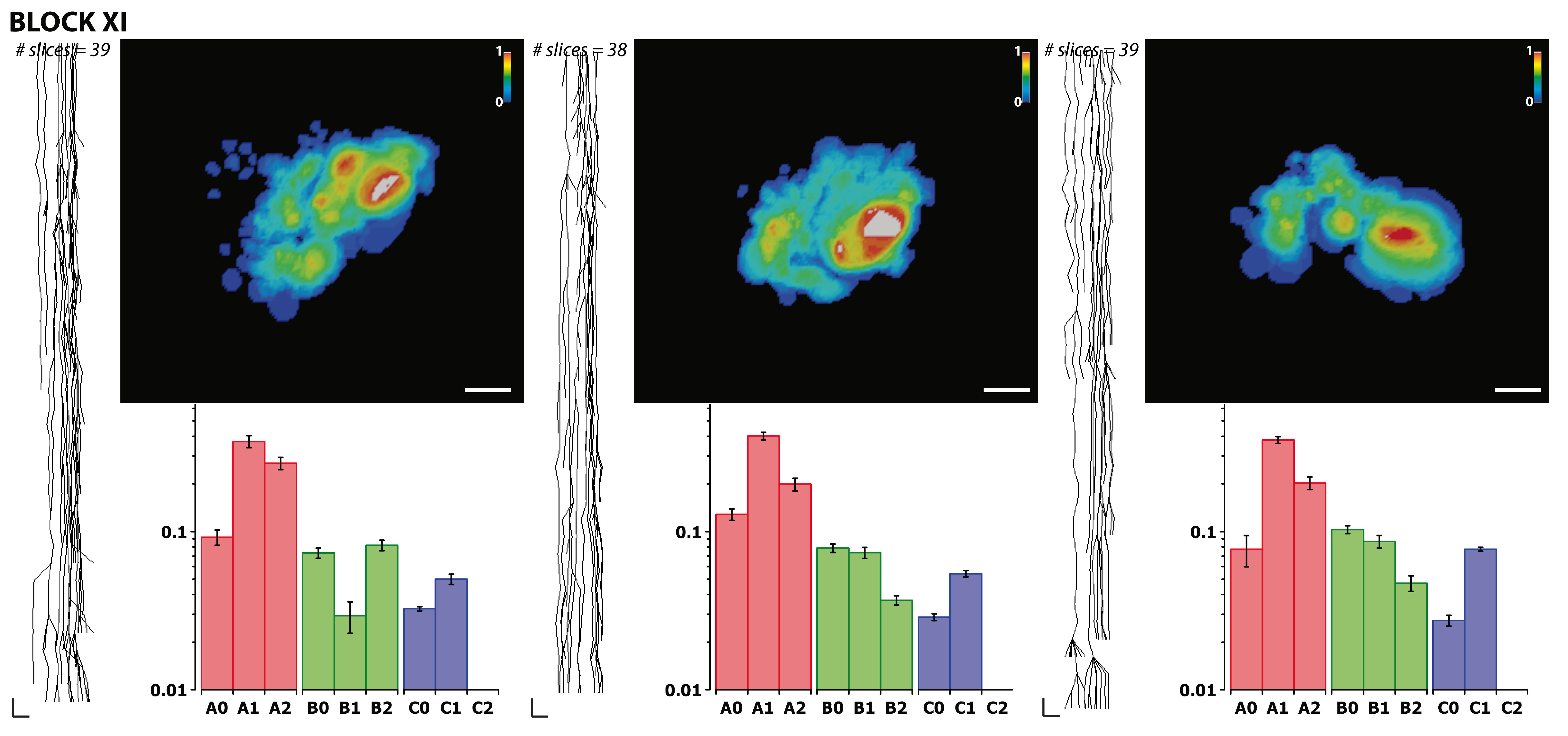

Supplement: Figure S12 — Reconstruction of each of the segments of the median nerve corresponding to block XI. See caption for Figure S2. [file Image12.JPEG]
