# Supplementary material for: Fascicular Topography of the Human Median Nerve for Neuroprosthetic Surgery
Source: Front Neurosci. 2016 Jul 1;10:286. doi: 10.3389/fnins.2016.00286 (PMC4929846; doi:10.3389/fnins.2016.00286)

LaTeX code for Supplementary Table S1

% Table S-1

\begin{table}[h!]

\begin{center}

\begin{tabular}{rc}

\textbf{Number of specimens} & \textit{5} \\

\\

\textbf{Length of median nerve} & \textit{71.3\textpm1.2 cm} \\

Upper arm & 29.1\textpm0.5 cm \\

Forearm & 25.6\textpm0.5 cm \\

Hand & 16.6\textpm0.7 cm \\

\\

\textbf{Number of branches forearm} & \textit{9.2\textpm1.1} \\

Muscular & 6.4\textpm0.4 \\

Sensory & 1.0\textpm0.0 \\

Articular & 2.0\textpm0.4 \\

\\

\textbf{Location} & \textit{\% of nFD {[}95\% confidence interval{]}} \\

sPT & 98.4\textpm2.5\% {[}91.45, 105.34{]} (n=5) \\

iPT & 88.2\textpm3.9\% {[}71.42, 104.98{]} (n=3) \\

Interosseous & 81.0\textpm3.0 \% {[}76.67, 89.32{]} (n=5) \\

FDS & 45.8\textpm4.3 \% (n=5) \\

Palmar cutaneous & 16.5\textpm6.6 \% (n=5) \\

\\

\boldmath$\mathrm{P_{M}(0.5)}$ & \textit{\% of nFD} \\

PL & 110.5\textpm11.1\% \\

ET & 104.5\textpm11.4 \% \\

PT & 95.7\textpm3.4 \% \\

FCR & 84.0\textpm4.5 \% \\

FDP, FPL, PQ & 78.5\textpm3.1 \% \\

FDS & 63.6\textpm8.3 \% \\

\end{tabular}

\\

\textbf{\refstepcounter{table}\label{S_table:01} Table S\arabic{table}.}{Summary of the anatomical values}

\end{center}

\end{table}


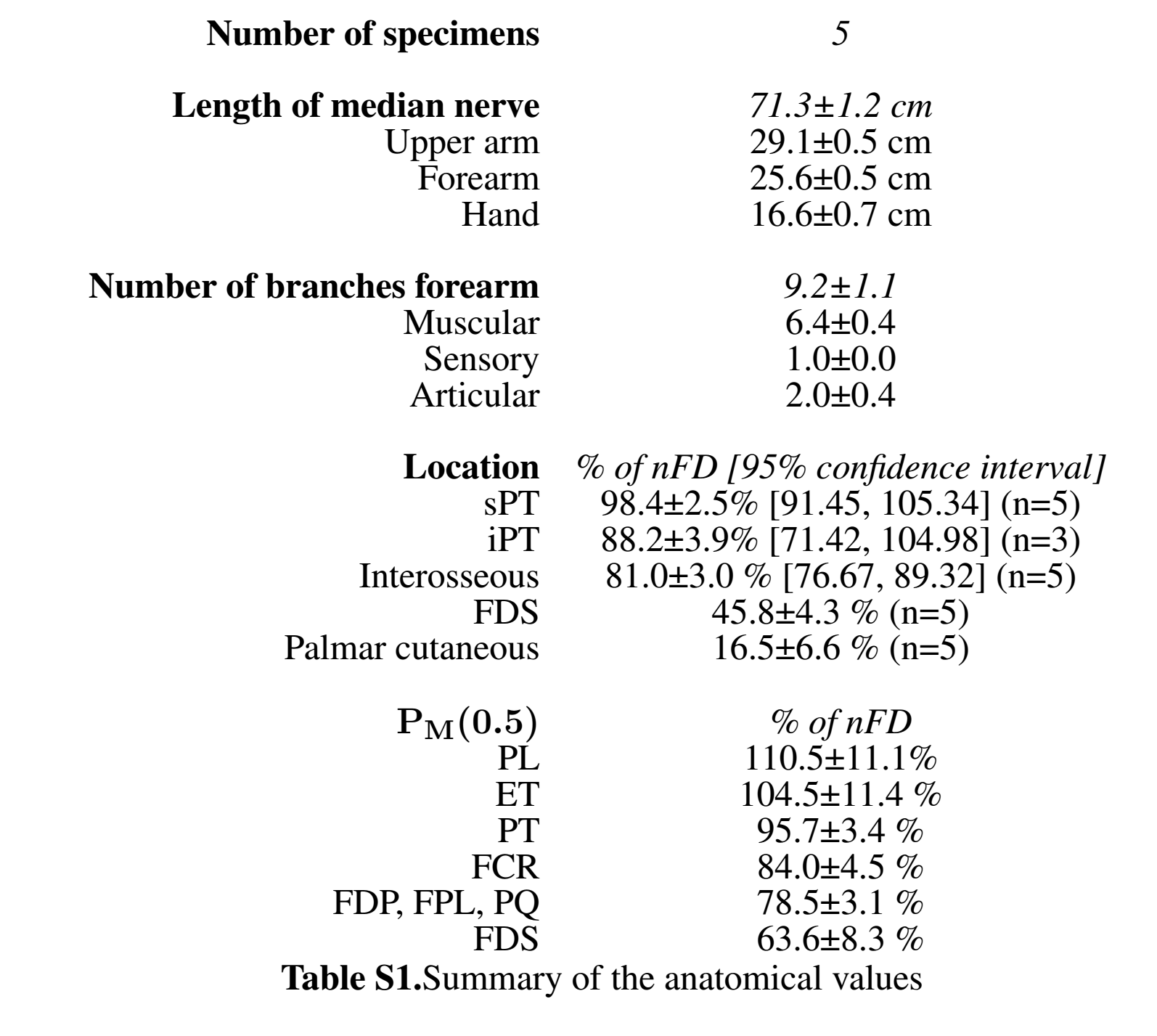

Supplement: Table S1 — Summary of the anatomical values. [file Table1.docx]
